# Perceived housing quality and emotional distress among refugees living in camps: a cross-sectional study in Eastern Germany

**DOI:** 10.1186/s12889-026-30396-8

**Published:** 2026-11-14

**Authors:** Soline Kulike, Lara Joanna Basekow, Carolin Schulenburg, Amand Führer

**Affiliations:** 1 Health Services Research Working Group, Interdisciplinary Center of Health Sciences, Medical Faculty of the Martin Luther University Halle-Wittenberg, Halle (Saale), Germany; 2 Institute of Psychology, Clinical Psychology and Psychotherapy, Faculty of Behavioral and Social Sciences, Chemnitz University of Technology, Chemnitz, Germany

**Keywords:** Refugees, Asylum seekers, Mental health, Housing quality, Refugee camp, Collective accommodation, Social determinants of health, Housing policy

## Abstract

**Background:**

Housing quality as a key social determinant of health has received growing attention in public health research. Asylum seekers and refugees in European countries are frequently housed in institutionalized, camp-like facilities. These facilities are often characterized by overcrowding and limited privacy, safety, and autonomy. Such conditions constitute an important post-migration stressor and may be a particularly relevant determinant of mental health. This study aimed to examine the association between multiple dimensions of perceived housing quality and emotional distress among refugees living in camps in Eastern Germany.

**Methods:**

We conducted a cross-sectional study among asylum seekers and refugees living in 16 camps across three federal states in Eastern Germany. Data were collected via a multilingual online survey; fieldwork took place in June and July 2025, and the survey remained accessible until the end of August 2025. As the primary outcome, we assessed emotional distress using the cross-culturally validated Refugee Health Screener-13 (RHS-13) as an indicator of common mental disorders in refugee populations. In addition, we assessed perceived housing quality using multidimensional indicators as well as relevant sociodemographic covariates. To examine associations between housing quality and emotional distress, we fitted multivariable linear regression models with housing quality indicators as exposures and the RHS-13 sum score as the outcome. First, each housing quality indicator was included separately in single-indicator regression models, which were adjusted for prespecified sociodemographic variables. We then included multiple indicators across different housing quality dimensions simultaneously in the main model, which was also adjusted for sociodemographic variables.

**Results:**

Most participants scored at or above the cutoff for emotional distress (79%; RHS-13 ≥ 11), with nearly half scoring in the severe-distress range (48%; RHS-13 ≥ 25). Most assessed housing quality indicators showed associations with emotional distress when examined separately in adjusted single-indicator regression models. When multiple indicators across multiple housing quality dimensions were included simultaneously in the adjusted main model, safety, lighting conditions, autonomy, and feelings of boredom or imprisonment within the refugee camp remained associated with emotional distress, whereas crowding, frequent transfers between camps, and region did not.

**Conclusions:**

Perceived housing quality is closely associated with emotional distress among refugees living in Eastern German camps. Psychosocial and institutional dimensions of housing quality appear particularly relevant to emotional distress. Housing policies for refugees in European countries should therefore consider not only the physical structure of housing but also its psychosocial and institutional dimensions, such as safety, autonomy, and daily-life restrictions, when addressing mental health inequalities in this population.

**Supplementary Information:**

The online version contains supplementary material available at https://doi.org/10.1186/s12889-026-30396-8.

## Background

### Housing as a multidimensional concept

The right to *adequate housing* is recognized as part of the right to an adequate standard of living, as established in the 1948 Universal Declaration of Human Rights [1] and reaffirmed in the 1966 International Covenant on Economic, Social, and Cultural Rights [2]. In clarifying what constitutes adequate housing, the UN Committee on Economic, Social, and Cultural Rights emphasized that this right should not be interpreted narrowly; drawing on the Committee’s General Comments, the Office of the UN High Commissioner for Human Rights and UN-Habitat state that “adequate housing must provide more than four walls and a roof” [3]. In the 21st century, this concept evolved into the broader term *healthy housing*, defined by the World Health Organization (WHO) as a shelter “[…] that provides a feeling of home, including a sense of belonging, security, and privacy” [4]. Collectively, these definitions highlight that housing is a complex and multifaceted concept, comprising both tangible dimensions, such as the physical structure of a shelter, and less tangible dimensions, including security, privacy, and control afforded by a home.

### Housing quality and health

The conceptual complexity of housing translates into challenges in measuring housing quality. Although the relationship between housing and health has been recognized since the emergence of modern public health [5], with researchers identifying multiple pathways through which housing affects health [6–10], contemporary frameworks remain challenged by the lack of global consensus on measurement standards of housing quality, limiting adequate estimation of housing-related health burden [11].

In this study, housing quality is understood as a multidimensional concept encompassing physical and structural conditions, as well as psychosocial and institutional aspects that shape residents’ everyday life [4, 12, 13]. It is thus narrower than living standards, which denote households’ material and economic situation, such as income, consumption, and wealth [2, 14], and living conditions, which encompass broader objective circumstances of life, including work, education, and health [15].

Tangible aspects of housing quality, such as room size or deterioration, can be measured objectively, for example through floor area or observer-rated instruments such as the Small-area Housing Environment Deterioration (SHED) index [16, 17]. Less tangible aspects, such as experienced safety and privacy, are harder to capture with standardized objective indicators because they depend partly on individual and contextual expectations [9]. The type of dimension is distinct from the measurement approach: room size, for example, can also be assessed subjectively by asking whether residents find their space adequate [13, 18]. This study focuses on perceived housing quality (see Methods). Importantly, the two approaches are complementary rather than interchangeable [13].

### Housing for asylum seekers and refugees

Across Europe, asylum seekers are commonly housed in collective accommodation during their asylum procedure, often with limited choice regarding their place of residence and the duration of their stay [10, 19]. In Germany, asylum seekers are not allowed to choose their place of residence. At the time of data collection for this study, they were generally required to live in a state-run initial reception facility until a decision on their application or, after a rejection, until departure, for a maximum of 18 months (six months for families with minor children), with exceptions in specific cases (Sect. 47 of the Asylum Act) [20]. Thereafter, they are allocated to a district or municipality and housed in collective accommodation there (Sects. 50 and 53); further transfers between facilities may nevertheless occur. Following a decision on their asylum application, they may move to private housing, although subject to varying regional regulations. Despite being intended as temporary, many refugees remain in collective accommodation centers for extended periods, as labor restrictions, limited income, and discrimination in the housing market create structural barriers to private housing [10].

The housing conditions in these centers are often described as substandard, inhumane [21], and unhomely, lacking essential features of a home, such as privacy, autonomy, and control over daily life [22]. A recent review highlights a growing trend toward *campization* of European refugee accommodation, characterized by large-scale, remote, and spatially demarcated camp-like facilities that consist of structures not originally intended for housing, such as containers or industrial buildings [19]. Evidence from multiple countries indicates that refugees living in such facilities often report feeling like “being in prison” [23–30].

Adequate and secure housing may be particularly important for the mental health of refugees, as experiences of war, infrastructure destruction, and forced displacement are well-known risk factors for mental ill health. Therefore, examining housing quality in refugee camps, including features that distinguish them from other housing forms, is imperative, as these may affect their mental health.

### Knowledge gaps and research questions

While qualitative research has provided valuable insights into refugee experiences in camps [31, 32], the impact of housing quality on mental health has not been sufficiently quantified. Most existing quantitative studies employ simplified or dichotomous classifications—such as stable versus unstable or shared versus private housing—or examine a single housing dimension [33, 34]. A recent quantitative study addressed this limitation by examining multiple dimensions of collective refugee accommodation [35]. However, it analyzed the combined impact of these dimensions as a cluster. A more differentiated analysis of how individual dimensions relate to mental health is still required. A significant challenge lies in operationalizing housing quality for refugee populations living in camps, which requires measurement standards based on consistent, clear definitions of quality indicators. Although the WHO provides a valuable synthesis of healthy housing indicators through its Housing and Health Guidelines (HHGL), it recognizes that this synthesis remains incomplete, as the guidelines do not distinguish between permanent and temporary housing [4], thereby limiting their applicability to refugee camps. For refugee populations, validated measurement frameworks and standardized quality indicators are particularly scarce, constraining adequate quantification and impeding a comprehensive understanding of housing quality as a social determinant of health.

Against this background, this study has two objectives: first, to broadly assess how refugees housed in camps in Eastern Germany perceive their housing quality using multidimensional indicators; and second, to examine the association between these indicators and emotional distress.

In this article, we use the term *camp* as an analytical category rather than an administrative designation [19, 36–38]. It refers to both the physical structure and the institutional practices that shape daily life in collective refugee accommodation, including initial reception facilities and municipal collective accommodation centers. Although these differ in legal basis and organization, they share, to varying degrees, key features such as spatial separation, institutional regulation of daily life, restricted autonomy and privacy, and imposed temporariness [19, 36–38]. We do not thereby equate these facilities with camps in other historical contexts, in particular concentration or internment camps. We are aware that the term may carry stigmatizing connotations; however, stigmatization arises primarily from the separation of residents rather than from naming it, and residents themselves frequently refer to these facilities as camps [19, 30]. The term thus characterizes the institutional setting, not its residents, who also exercise agency within it [19, 38]. In addition, we use the term *refugee* to include all refugees, asylum seekers, and displaced persons in refugee-like situations.

## Methods

### Study design

This study collected primary data via a multilingual online survey across three federal states in Eastern Germany (Brandenburg, Mecklenburg-Western Pomerania, and Saxony-Anhalt). Fieldwork took place from June 20 to July 10, 2025; thereafter, the survey remained accessible until the end of August 2025 so that residents absent during the visits could participate. A cross-sectional design was selected because it is well-suited for estimating prevalences and examining associations between exposure indicators and outcomes without implying causality [39]. An online survey was selected for feasibility, as prior work suggests that technology-based self-administered questionnaires can be acceptable in socially disadvantaged and low-literacy populations [40, 41]. This study was part of the “Overcoming Barriers – Psychosocial Care of Refugees” project. Further findings from this project, concerning healthcare access and loneliness among refugees, will be published separately.

Germany is a relevant setting for examining refugee housing quality. In 2025, it received the highest number of applications for international protection in the EU+ [42]. Its camp-based reception system shares key features, such as mandatory residence in collective facilities, spatial segregation, and institutional regulation of daily life, with those of many other European countries [19]; findings from Germany may therefore be informative for comparable settings. The three federal states were selected because they were part of the “Overcoming Barriers” project region. Within these states, including rural camps was particularly relevant because research on refugee reception and integration in rural settings remains comparatively limited [43].

### Sampling

We employed a stratified cluster sampling design covering both urban and rural regions in the participating federal states. Urban regions were defined as large cities with at least 100,000 inhabitants; regions with fewer than 100,000 inhabitants were classified as rural. Eligible urban and rural camps were selected separately in each participating state. Camps were eligible if they housed at least 150 non-Ukrainian residents. This pragmatic threshold reflected resource constraints, as conducting on-site visits to numerous small facilities across three states would have yielded few participants per visit. Ukrainian refugees were excluded because this study focused on camp-specific housing exposures. Under the EU Temporary Protection Directive, they receive a residence permit without undergoing an asylum procedure, are not required to live in initial reception facilities [44–46], and mostly live in private housing [47].

In the first step, we aimed to obtain comprehensive lists of refugee camps in each participating state. Since no state-level camp registers were available for this study, we contacted all district authorities from February to May 2025 to request information on camp locations, facility operators, capacities, current occupancies, and residents’ nationalities and native languages. Despite follow-up emails and calls, not all authorities responded, and some declined to provide the requested information, citing data protection concerns or seeing no need to participate. Consequently, the sampling frame was restricted to camps in regions that provided the requested lists and comprised 55 eligible camps (7 urban, 48 rural); each listed camp was assigned a separate number.

The sampling strategy aimed to achieve a broadly balanced representation of urban and rural settings while ensuring regional heterogeneity. However, due to the limited availability of eligible urban camps, the selection procedure had to be adapted. Because few urban camps were available (none in Brandenburg and one in Saxony-Anhalt), we included all camps in the only participating urban region in Mecklenburg-Western Pomerania that responded to us. In the rural stratum, all eligible camps were enumerated and assigned unique identifiers, and two camps were randomly selected per state; in Brandenburg, where no urban camp could be included, one additional rural camp was drawn. In Mecklenburg-Western Pomerania, each of the two drawn camps was located on a site with a second unit run by the same operator; these camp units were visited and included. The asymmetry between the sampling of rural and urban camps reflects the available sampling frame rather than the intended design.

The final sample encompassed 16 camps: 10 in Mecklenburg-Western Pomerania (six urban, four rural), three in Brandenburg (all rural), and three in Saxony-Anhalt (one urban, two rural). The total reported occupancy of these camps was approximately 4,000 residents.

### Data collection and participants

Prior to data collection, participating camps received posters in all survey languages (see below). These posters informed residents about the study’s objectives, its independence from government authorities, and the voluntary and anonymous nature of participation. The posters also included fieldwork dates and the study team’s contact details. Camp staff were asked to display these posters in communal rooms accessible to all residents.

To ensure standardized implementation during data collection, the study team held a preparatory seminar to discuss questionnaire content, ethical considerations, and fieldwork procedures. The multilingual study team consisted of eight members, including medical professionals and graduate students from medicine, psychology, and anthropology. Three team members had experience as linguistic and cultural mediators for refugees in psychosocial contexts.

Fieldwork was conducted between June 20 and July 10, 2025, with our study team visiting each camp in person. Visits took place on weekdays and, in one instance, on Saturday at different times of day (eight camps were visited before noon and eight after noon, with the latest visits beginning in the late afternoon). Recruitment was planned as door-to-door outreach, with on-site camp staff accompanying the study team to residents’ rooms without entering them. Residents were approached at their room doors, and eligible residents received leaflets in their respective languages. These leaflets contained written information on the study’s objectives, its independence from government authorities, and the anonymity of participation. After providing informed consent, participants could access the survey via a QR code printed on the leaflets, either during the visit or later. Participants were informed that the study team was available on-site during the visit to answer questions if needed. To reduce access-related barriers, mobile devices and internet access were provided by the study team as needed.

At several sites, camp staff denied our team access to the room floors and instead organized group meetings with available residents in communal rooms. As camp staff were occasionally present during survey completion, participants were instructed to complete the questionnaire without others viewing their responses and without group discussions with other participants. Participants with limited literacy could complete the survey as an interview, in which a team member read the questions aloud and recorded the answers; the number of such interviews was not recorded. No incentives were offered for participation.

Residents were eligible for participation if they met the following criteria: (1) at least 16 years old, (2) housed in one of the selected camps, (3) not of Ukrainian nationality, and (4) reporting sufficient proficiency in at least one of the survey languages.

Non-participation was not systematically recorded, as no reliable resident lists were available, occupancy fluctuated daily, and documenting non-consenting residents would have conflicted with our anonymity guarantees. Relative to the total reported occupancy of approximately 4,000 residents, including ineligible residents, the 410 participants represented approximately 10% of reported occupancy; this figure does not constitute a formal response proportion because the eligible denominator was unknown. Field notes documented reasons for non-participation, including absence, staff-imposed access restrictions, and personal refusal. Reasons noted for personal refusal included concerns about residence status or potential conflicts with camp staff, perceived lack of benefit from participation, and competing personal priorities. To enhance participation, copies of the study leaflets were left at all visited camps. This enabled residents who were absent during our team’s visit to participate later.

### Questionnaire development

The questionnaire comprised 114 items across five thematic sections: perceived housing quality, mental health conditions, loneliness, healthcare access, and sociodemographic characteristics. It included cross-culturally validated instruments, adapted items from instruments validated in other populations, and self-developed items. Self-developed items were formulated in German, while all adopted instruments were originally available in English. Based on information from camp managers about residents’ most common native languages, the questionnaire was translated into English, Arabic, Persian (Farsi), Turkish, French, Kurdish (Kurmanji), and Pashto, as applicable. A certified translation agency performed these translations following standardized forward-backward translation procedures. For the RHS-13, existing validated language versions were used for Arabic, Persian, English, French, and Pashto, whereas the Kurdish (Kurmanji) and Turkish versions were newly translated. Additional file 1 documents the original language and translations of each item. Our multilingual study team reviewed all translations to ensure conceptual and linguistic equivalence.

To reduce respondent burden due to the large number of items, filter logic was implemented throughout the survey (e.g., only participants who reported sharing their room were asked to rate the crowding in the room).

### Measurement and operationalization

Detailed information on all questionnaire items used in this study is provided in Additional file 1. This includes wording, codes, sources, adaptations, translations, and proportions of missing data. This section focuses on variables that were further transformed or were particularly relevant for statistical analyses.

#### Sociodemographic variables

Response options for sociodemographic variables reported by fewer than 10 participants were grouped into *other* subcategories. For gender, data for the extremely small proportion of participants identifying as *diverse* were recorded as missing to protect confidentiality. Length of stay in Germany was defined as the number of months between arrival in Germany and the date of data collection.

Participants’ dependency on language interpreters in everyday life was self-reported through a binary variable (no vs. yes). Subjective general health status was measured using the first item of the Short-Form Health Survey (SF-12) [48] and additionally dichotomized into *good* (reference) for *excellent/very good/good*, and *poor* for *fair/poor*.

#### Mental ill health

The present study focused on emotional distress as an indicator of mental ill health. Emotional distress was assessed using the Refugee Health Screener-13 (RHS-13), a shortened version of the RHS-15, which was empirically developed to screen for common mental disorders in heterogeneous refugee populations [49, 50]. The instrument uses 13 items to screen for symptoms of depression, anxiety, post-traumatic stress disorder (PTSD), and somatization. However, results are not intended to differentiate specific mental disorders. An additional benefit of the RHS-13 is that it does not include questions about specific traumatic events. This is particularly advantageous given the re-traumatization risk associated with mental health screening [51].

Items are rated on a 5-point scale (0 = *not at all* to 4 = *extremely*) reflecting symptom burden over the past month, yielding a total sum score ranging from 0 to 52, with higher scores indicating greater emotional distress. At the time of questionnaire development, 21 language versions of the RHS-13 had been cross-culturally validated and were available free upon request.

In a 2017 study of a refugee sample in Germany, the RHS-13 demonstrated excellent psychometric properties (Cronbach’s α = 0.91 and 0.93, sensitivity = 0.95 and 0.79, specificity = 0.87 and 0.78 for the interview-administered and self-report versions, respectively, at a cutoff score of 11) [52]. A validation study in Sweden confirmed this cutoff score for identifying individuals in need of further clinical assessment and proposed additional thresholds to distinguish between mild (11–17), moderate (18–24), and severe (≥ 25) levels of distress as guidance for clinical decision-making [53].

As the RHS-13 constituted the primary outcome measure, all RHS-13 items were mandatory; that is, only participants who completed all 13 items were able to continue the survey.

Based on the established cutoff scores, three variables were derived: RHS-13 score (continuous; sum score), screening result (binary; *negative* < 11/*positive* ≥ 11), and distress severity (ordinal; *no distress/mild/moderate/severe distress*). The binary and ordinal variables were used only for descriptive reporting of prevalence and severity of emotional distress; all regression analyses used the continuous sum score.

#### Perceived housing quality

To facilitate interpretation and provide a clear analytical structure, each housing quality indicator assessed in the present study was mapped to a corresponding housing theme derived from previous conceptual frameworks [12, 13]. Table 1 presents an overview of this mapping, which served as the theoretical basis for our operationalization of housing quality. The indicators primarily captured participants’ subjective perceptions of their housing quality, complemented by self-reported structural and institutional housing dimensions. This focus reflected our study’s interest in residents’ lived experience in relation to emotional distress. Additional observer-rated and objective measurements were not conducted because access to private rooms was limited and room-level measurements could not readily be linked to anonymous survey responses.

**Table 1:** Mapping of items and indicators to housing themes

Psychosocial attributes from Dunn, 2002 [12]	Housing theme from Brake et al., 2023 [13]	Indicators from the present study	Response options (analytic categories)
Control	Crowding, space, and privacy	Crowding (camp/room/kitchen/shower/toilet)	Extremely intolerable/intolerable/tolerable/adequate (adequate; tolerable; (extremely) intolerable)
		Room size	Very good/fairly good/rather bad/very bad (good; poor)
		Residential constellation in own room	Alone/with family/with friends or acquaintances/with other residents
		Possibility to lock own room	Yes/no
		Possibility to store personal items in lockable cabinets in own room	Yes/no
		Availability of own kitchen/shower/toilet	Kitchen: yes/no; shower and toilet: own/shared with same-sex residents/shared with residents of different sexes/none
		Provision with food or shared kitchens	Yes/no
Control	Housing instability, including security of tenancy, affordability, and residential mobility	Number of transfers between camps	0/1/2/3/4/5/> 5 (frequent transfers: no < 3; yes ≥ 3)
Control	Safety	Personal safety (camp/room/shower/toilet)	Very safe/rather safe/rather unsafe/very unsafe (safe; unsafe)
	Physical conditions of/in the dwelling	Lighting conditions (room)	Very good/fairly good/rather bad/very bad (good; poor)
		Hot water supply (camp)	Very good/fairly good/rather bad/very bad (good; poor)
		Hygiene conditions (shower/toilet/kitchen)	Very good/fairly good/rather bad/very bad (good; poor)
	Satisfaction	–	–
Demand	Neighborhood/location	Place of residence	List of visited camps (region: urban; rural)
Control & Expressing status	Institutional practices	Room inspections	Yes/no
		Central food provision (e.g., daily catering)	Yes/no
		Quality and quantity of served food	Very good/fairly good/rather bad/very bad (good; poor)
		Participation and autonomy in daily life	Very good/good/fair/bad/very bad (mean score across nine items, 1–5)
		Frequency of feeling bored	Never/rarely/sometimes/often/always (low: never–sometimes; high: often/always)
		Frequency of feeling imprisoned	Never/rarely/sometimes/often/always (low: never/rarely; high: sometimes–always)
		Separation from spouse and/or children in the camp	Spouse: yes/no; children: all/some/none of the children in the same camp

Response options are shown as presented in the questionnaire; categories used in the regression analyses are given in parentheses. Item wording, item codes, and sources are provided in Additional file 1

Response options for crowding measurement were adopted from crowding research in public transportation [54]. Autonomy in refugee camps was conceptualized as residents’ capacity to self-determine when and how they engage in daily activities according to their preferences. To measure autonomy, we used the Autonomy Indoors and Family Role domains of the Impact on Participation and Autonomy Questionnaire (IPAQ) [55]. We adapted three items contextually and additionally developed two items following the scale’s phrasing conventions.

The IPAQ was originally developed to assess participation and autonomy among individuals with chronic disorders, emphasizing perceived needs and experiences rather than functional limitations. It includes 31 items rated on a 5-point scale (1 = *very good* to 5 = *very poor*) across five domains. In a validation study on rehabilitation patients in the Netherlands, the Autonomy Indoors and Family Role domains demonstrated good homogeneity (Cronbach’s α = 0.91 and 0.90, respectively) [56]. Additionally, a mean-score index was constructed from the nine items used, with higher scores indicating greater restrictions.

Further institutional dimensions of housing included frequent feelings of boredom and imprisonment, assessed with two items referring explicitly to experiences in the camp over the past two weeks. Drawing on qualitative research on refugee camps, boredom was conceptualized as an expression of enforced inactivity due to labor restrictions, lack of meaningful activities, and prolonged waiting [23, 24, 30, 31, 57], and feelings of imprisonment as an expression of restricted mobility, surveillance, and spatial segregation [19, 23, 26, 58, 59]. For boredom, never/rarely/sometimes were combined into low (reference), whereas for feeling imprisoned, only never/rarely were combined into low (reference), as we considered occasional boredom relatively common in camp settings, whereas occasional feelings of imprisonment were considered adverse.

Transfers between camps were operationalized from an item asking residents how often they had to be transferred since arriving in Germany (0, 1, 2, 3, 4, 5, or > 5). The item did not distinguish permanent relocations from temporary stays, nor did it capture reasons for transfers, which may include routine reallocation from state to municipal responsibility, sanctions, or allocation of newly arriving residents. We classified three or more reported transfers as frequent.

### Statistical analyses

All statistical analyses were conducted in R version 4.5.1 using RStudio version 2025.09.0. Data wrangling and cleaning were performed using core tidyverse packages [60]. Missing data increased toward the end of the questionnaire (from up to 15% for housing quality items and approximately 16% for RHS-13 items to 18–27% for subsequent health and sociodemographic items; Additional file 1), a pattern consistent with missingness being driven largely by survey discontinuation as the questionnaire progressed. We therefore considered a missing-at-random (MAR) assumption plausible [61] and handled missing data using multiple imputation by chained equations (mice package [62], 10 imputations, 5 iterations, seed = 1234). Estimates were pooled across imputed datasets according to Rubin’s rules. Imputed data were used only for regression analyses; descriptive statistics were based on non-imputed data following an available-case approach. Linear regression models were fitted using the lm() function of the base R stats package. Using the gtsummary package [63], we calculated frequencies and proportions for categorical variables and minimum, maximum, mean, and standard deviation for continuous variables. The number of available cases per item is reported accordingly.

For regression analyses, response options for all ordinal variables were semantically grouped into combined categories to ensure sufficient cell sizes and avoid overfitting. Reference categories were selected consistently across variables: when no inherent semantic ordering was assumed (e.g., gender, religion), the largest category served as the reference. For variables contrasting positive versus negative characteristics (e.g., room inspections), the positive category served as the reference.

In the first step of regression analyses, we included all housing quality indicators in separate models, with dummy-coded combined categories as predictors and the RHS-13 score as the dependent variable. Each of these models (hereafter referred to as single-indicator models) was adjusted for age, gender, country of origin, marital status, length of stay in Germany, dependency on language interpreters, and subjective general health status. These covariates were selected a priori as potential confounders based on previously reported associations with mental health [64, 65]. Because the objective was to estimate associations adjusted for the prespecified confounder set rather than to decompose the incremental contribution of covariate blocks, all covariates were entered simultaneously. Visual inspection of residual diagnostic plots from representative imputed datasets indicated approximately normally distributed residuals. Although no pronounced heteroscedasticity was apparent visually, Breusch–Pagan tests indicated evidence of heteroscedasticity in nine of the ten imputed datasets. As the distribution of the outcome showed no pronounced skewness and the residual distribution was approximately normal, no transformation of the RHS-13 sum score was applied. Heteroscedasticity was further examined in a robustness analysis using HC3 heteroscedasticity-consistent standard errors (see below).

In the next step, we analyzed indicators from all housing quality dimensions within a single regression model. To avoid overfitting and minimize multicollinearity, we included a single representative variable for each housing quality dimension assessed across multiple spatial contexts (e.g., room, kitchen, shower, toilet). To ensure comparability across all participants, we only included variables assessing general spatial contexts accessible to all residents. These variables were derived from survey items that did not rely on filter logic and were therefore received by all participants (main model; *n* = 309). We prioritized variables that we hypothesized to be particularly relevant to perceived vulnerability and mental health, while ensuring balanced coverage across all spatial contexts of the camp. All adjusted generalized variance inflation factors in the main model were below 1.73, suggesting no substantial multicollinearity. The main model estimated 21 coefficients, including the intercept (20 predictor coefficients), based on 309 observations, corresponding to approximately 15.5 observations per predictor coefficient. Due to the exploratory nature of this study, no a priori sample size calculation was performed.

Finally, we conducted sensitivity and robustness analyses. To assess the impact of multiple imputation, we refitted the main model using non-imputed complete-case data (*n* = 276). To examine potential overadjustment, we refitted the main model without subjective general health status and dependency on interpreters. In addition, because Breusch–Pagan tests indicated heteroscedasticity, we performed a robustness analysis using heteroscedasticity-consistent HC3 standard errors. For multiply imputed analyses, HC3 variance estimates were calculated separately within each imputed dataset and pooled according to Rubin’s rules. Model diagnostics and results of the HC3 robustness analysis are provided in Additional file 4.

## Results

The base analytical sample comprised 410 participants. Of these, 343 completed the RHS-13, and 316 completed the full questionnaire. Thus, 67 participants discontinued before completing the RHS-13, while a further 27 discontinued thereafter.

### Descriptive statistics

#### Sociodemographic characteristics

Most participants were male (80%, *n =* 255), with an average age of 31 years. Almost half of the participants were aged 25 to 35 years (46%, *n =* 145). The most common countries of birth were Syria (31%, *n =* 99) and Afghanistan (19%, *n =* 59). Length of stay in Germany ranged from 1 to 204 months, with the largest proportion reporting stays of 1 to 2 years (32%, *n =* 101) and the smallest proportion reporting stays exceeding 10 years (1.3%, *n =* 4).

Most participants were single (58%, *n =* 186) and reported having no children (59%, *n =* 188). Most reported Islam as their religious affiliation (80%, *n =* 240). More than two-thirds reported dependency on interpreters in everyday life (68%, *n =* 216), and over half rated their general health as *poor* (fair/poor) (56%, *n =* 188). Table 2 presents detailed sociodemographic characteristics of our sample.

**Table 2:** Sociodemographic characteristics of survey participants

	Available cases	% (*n*) or Min., Max., Mean (SD)
Gender	319	
Male		80 (255)
Female		20 (64)
Age (years)	318	16, 70, 31 (10)
Age group	318	
16 to < 25 years		23 (74)
25 to < 35 years		46 (145)
35 to < 45 years		22 (70)
≥ 45 years		9.1 (29)
Country of origin	318	
Syria		31 (99)
Afghanistan		19 (59)
Benin		6.9 (22)
Iran		6.9 (22)
Turkey		6.9 (22)
Iraq		4.4 (14)
Somalia		3.5 (11)
Other African countries^a^		16 (50)
Other Asian countries^b^		4.7 (15)
Other countries		1.3 (4)
Length of stay in Germany *(months)*	312	1, 204, 21 (23)
Length of stay in Germany *(categorized)*	312	
0 to < 6 months		13 (40)
6 to < 12 months		24 (76)
1 to < 2 year(s)		32 (101)
2 to < 5 years		26 (82)
5 to < 10 years		2.9 (9)
≥ 10 years		1.3 (4)
Federal state of residence	360	
Mecklenburg-Western Pomerania		58 (209)
Brandenburg		23 (81)
Saxony-Anhalt		19 (70)
Region	360	
Urban		45 (162)
Rural		55 (198)
Marital status	320	
Married		35 (112)
Single		58 (186)
Divorced		5.0 (16)
Widowed		1.9 (6)
Living with spouse in Germany	112	
Yes		37 (41)
No		63 (71)
Children	319	
No		59 (188)
Yes		41 (131)
Living with children in Germany	131	
All children are living in Germany		44 (57)
Not all children are living in Germany		12 (16)
All children are not living in Germany		44 (58)
Religious affiliation	301	
Islam		80 (240)
Christianity		13 (39)
None		6.3 (19)
Other religion		1.0 (3)
Dependency on interpreters in everyday life	319	
No		32 (103)
Yes		68 (216)
Subjective general health status	334	
Excellent		9.0 (30)
Very good		9.0 (30)
Good		26 (86)
Fair		31 (104)
Poor		25 (84)

Percentages may not add up to 100 due to rounding. ^a^Other African countries: Algeria, Cameroon, Egypt, Gambia, Ghana, Guinea-Bissau, Kenya, Mali, Mauritania, Nigeria, Sierra Leone, South Africa, Sudan^b^Other Asian countries: Armenia, India, Pakistan, Palestine, Russia, Tajikistan

#### Prevalence of emotional distress

Among the 343 participants who completed the RHS-13, sum scores ranged from 0 to 52 (*M* = 23, *SD* = 13). Most participants (79%, *n =* 271) scored at or above the established RHS-13 cutoff of 11, indicating emotional distress. In total, 16% (*n =* 56) scored in the mild-distress range, 15% (*n* = 51) in the moderate-distress range, and nearly half (48%, *n* = 164) in the severe-distress range.

Descriptively, women showed higher levels of emotional distress than men on average (women: *M* = 26, *SD* = 14, 95% CI [23, 30]; men: *M* = 22, *SD* = 3, 95% CI [21, 24]) (Table 3). This difference was not formally tested, and the confidence intervals overlap; however, female gender remained associated with higher distress in the adjusted main model (see below).

**Table 3:** Prevalence of emotional distress in study population by gender

	Statistic	Male *N* = 255	Female *N* = 64	Overall *N* = 319
RHS-13 score	min., max., M [95% CI]	0, 52, 22 [21, 24]	0, 51, 26 [23, 30]	0, 52, 23 [22, 25]
RHS-13 screening result				
Negative	% (*n*) [95% CI]	23 (58) [18, 28]	17 (11) [9.3, 29]	22 (69) [17, 27]
Positive	% (*n*) [95% CI]	77 (197) [72, 82]	83 (53) [71, 91]	78 (250) [73, 83]
Severity of distress				
No distress	% (*n*) [95% CI]	23 (58) [18, 28]	17 (11) [9.3, 29]	22 (69) [17, 27]
Mild	% (*n*) [95% CI]	16 (42) [12, 22]	13 (8) [5.9, 24]	16 (50) [12, 20]
Moderate	% (*n*) [95% CI]	16 (42) [12, 22]	11 (7) [4.9, 22]	15 (49) [12, 20]
Severe	% (*n*) [95% CI]	44 (113) [38, 51]	59 (38) [46, 71]	47 (151) [42, 53]

RHS-13 cutoff scores used were: < 11 for no distress, 11–17 for mild distress, 18–24 for moderate distress, and ≥ 25 for severe distress. Percentages may not add up to 100 due to rounding. The table includes participants with complete RHS-13 and gender data (*N* = 319)

#### Perceived housing quality

Additional file 2 provides detailed descriptive statistics for all housing quality indicators, including stratified analyses by distress severity.

Overall, most participants rated crowding in all assessed spatial contexts as tolerable. Only a minority rated the number of residents sharing one kitchen, shower, or toilet as adequate, while crowding in one’s own room and in the camp in general were more commonly rated as adequate. Regarding available space, the largest proportion rated their room size as fairly good (44%, *n* = 158), while the availability of personal kitchens (9%, *n* = 36), showers (15%, *n* = 56), and toilets (13%, *n* = 52) was low. Regarding privacy, most reported being able to lock their rooms (94%, *n* = 359) and store personal items in lockable cabinets (66%, *n* = 251).

Regarding housing instability, only 12% (*n =* 38) reported no transfers between camps since arriving in Germany. Over half (55%, *n =* 173) reported one or two transfers, and a third (33%, *n =* 105) reported three or more transfers.

Most participants felt safe across all assessed spatial contexts, with the highest safety ratings reported for one’s own room.

Lighting conditions and hot water supply received the most favorable ratings of all indicators, with most participants rating them as good. Participants more frequently reported good hygiene conditions for kitchens and showers than for toilets.

Perceived participation and autonomy varied across indicators. Most participants perceived low autonomy regarding their ability to retreat to a private area and get minor repairs done in their rooms. Half reported poor chances of doing laundry when they wished or needed. In contrast, participants reported the highest perceived autonomy for practicing religious or cultural traditions. Regarding further institutional dimensions, high frequencies of feeling bored or imprisoned were reported for the past two weeks. Room inspections were also common. Centralized daily food provision (e.g., catering) was uncommon. Among those receiving centralized meals, food quality was more frequently rated as *poor* than food quantity.

### Multivariable regression analyses

#### Single-indicator regression models

Table 4 shows the coefficients and 95% CIs of all single-indicator models. Across all assessed spatial contexts, participants who perceived the number of co-residents they had to share a space with as *(extremely) intolerable* showed higher RHS-13 scores than those who rated it as *adequate*. *Tolerable* crowding was only associated with higher distress in the context of shared kitchens. Reporting poor room size, lighting, or hygiene conditions was also associated with increased distress, compared to reporting good conditions. Similarly, feeling unsafe across all assessed spatial contexts was associated with higher RHS-13 scores than feeling safe. In contrast, neither being able to store personal items in lockable cabinets nor experiencing room inspections by staff was associated with the RHS-13 score.

**Table 4:** Single-indicator regression models for selected housing quality indicators

	β	95% CI
Crowding (room) (ref. Adequate)		
Tolerable	2.5	[− 1.0, 6.0]
(Extremely) Intolerable	5.1	[1.8, 8.4]
Crowding (camp) (ref. Adequate)		
Tolerable	0.23	[− 2.9, 3.3]
(Extremely) Intolerable	6.1	[2.9, 9.2]
Crowding (kitchen) (ref. Adequate)		
Tolerable	6.7	[3.0, 10]
(Extremely) Intolerable	10	[6.7, 14]
Crowding (shower) (ref. Adequate)		
Tolerable	3.5	[− 0.71, 7.8]
(Extremely) Intolerable	7.5	[3.4, 12]
Crowding (toilet) (ref. Adequate)		
Tolerable	3.0	[− 1.2, 7.2]
(Extremely) Intolerable	6.9	[3.0, 11]
Room size Poor (ref. Good)	7.0	[4.5, 9.4]
Possibility to store personal items No (ref. Yes)	0.37	[− 2.3, 3.1]
Possibility to lock own room No (ref. Yes)	0.45	[− 4.7, 5.6]
Frequent transfers Yes (ref. No)	3.9	[1.2, 6.6]
Safety (room) Unsafe (ref. Safe)	8.7	[5.6, 12]
Safety (camp) Unsafe (ref. Safe)	7.5	[4.6, 10]
Safety (shower) Unsafe (ref. Safe)	6.9	[4.2, 9.6]
Safety (toilet) Unsafe (ref. Safe)	7.0	[4.2, 9.8]
Lighting conditions (room) Poor (ref. Good)	8.9	[5.7, 12]
Hot water supply (camp) Poor (ref. Good)	3.4	[− 1.6, 8.4]
Hygiene (kitchen) Poor (ref. Good)	6.3	[3.6, 9.0]
Hygiene (shower**)** Poor (ref. Good)	5.2	[2.6, 7.8]
Hygiene (toilet) Poor (ref. Good)	5.9	[3.5, 8.4]
Region Rural (ref. Urban)	2.7	[0.14, 5.2]
Autonomy (mean score)	5.5	[4.2, 6.8]
Room inspections Yes (ref. No)	−0.28	[− 3.0, 2.4]
Frequency of feeling bored High (ref. Low)	8.1	[5.5, 11]
Frequency of feeling imprisoned High (ref. Low)	11	[8.1, 13]

All models are adjusted for age (years), gender (ref. male), country of origin (ref. Syria), marital status (ref. single), length of stay in Germany (months), dependency on interpreters (ref. no), and subjective general health status (ref. excellent/very good/good). Estimates are pooled across 10 multiple imputed datasets using Rubin’s rule

#### Main regression model

Table 5 shows the results of the main regression model. Neither *tolerable* nor *(extremely) intolerable* crowding in the camp in general was associated with the RHS-13 score. However, poor room size remained associated with higher emotional distress compared to good room size. Frequent transfers between camps were not associated with the RHS-13 score in the main model. Feeling unsafe in one’s own room remained associated with increased distress compared to feeling safe, as did poor lighting conditions compared to good conditions. Being housed in rural regions was not associated with higher distress than being housed in urban regions. Among institutional dimensions, higher average scores of perceived participation and autonomy, indicating greater restrictions across the nine autonomy items, as well as higher frequencies of feeling bored or imprisoned, were associated with increased distress. Among the sociodemographic variables, being female, married, and of Afghan origin was associated with higher emotional distress compared to being male, single, and of Syrian origin. Age, length of stay in Germany, region, and dependency on interpreters were not associated with distress. Subjective general health showed the strongest overall association with emotional distress in the main model.

**Table 5:** Main regression model

	β	95% CI
Room size Poor (ref. Good)	2.51	[0.15, 4.87]
Crowding (camp) (ref. Adequate)		
Tolerable	−0.84	[− 3.52, 1.84]
(Extremely) Intolerable	0.05	[− 2.86, 2.95]
Frequent transfers Yes (ref. No)	0.96	[− 1.40, 3.32]
Safety (room) Unsafe (ref. Safe)	3.02	[0.18, 5.87]
Lighting conditions Poor (ref. Good)	5.01	[1.94, 8.08]
Region Rural (ref. Urban)	1.43	[− 0.72, 3.57]
Autonomy Indoors (mean score)	2.90	[1.55, 4.26]
Frequency of feeling bored High (ref. Low)	3.22	[0.64, 5.79]
Frequency of feeling imprisoned High (ref. Low)	4.69	[1.89, 7.49]

The model was adjusted for age (years), gender (ref. male), country of origin (ref. Syria), marital status (ref. single), length of stay in Germany (months), dependency on interpreters (ref. no), and subjective general health status (ref. excellent/very good/good). Estimates are pooled across 10 multiple imputed datasets using Rubin’s rule. Pooled adjusted R² = 0.54, 95% CI [0.47, 0.62], fmi = 0.01

#### Sensitivity and robustness analyses

Sensitivity analyses yielded results largely consistent with the main analysis. Refitting the main model with non-imputed complete-case data yielded results consistent in direction and magnitude with those from the imputed data. In this analysis, the 95% confidence interval for room size included the null (β = 2.1, 95% CI [− 0.44, 4.6]), and being married was not associated with emotional distress. Refitting the main model without subjective health status and dependency on interpreters yielded slightly larger effect estimates, while the overall direction and magnitude of associations remained broadly consistent with the main model. Detailed results of the sensitivity analyses are provided in Additional file 3. The HC3 robustness analysis likewise yielded results largely consistent with the primary analysis, with a slight decrease in precision for a few variables. In the main model, the association with poor room size was less precise, and its 95% confidence interval included the null (β = 2.51, 95% CI [− 0.12, 5.13]). The remaining overall pattern of associations was largely unchanged. Detailed results of model diagnostics and HC3 robustness analyses are provided in Additional file 4. Overall, the sensitivity and robustness analyses supported the main pattern of findings.

## Discussion

### Main findings

#### Prevalence of emotional distress

Our study revealed a high mental health burden among refugees housed in Eastern German camps. The vast majority of participants (79%) scored at or above the established RHS-13 cutoff for emotional distress, indicating symptoms of depression, anxiety, PTSD, and somatization. Nearly half of our sample scored in the severe-distress range, indicating a high symptom burden. This high prevalence is consistent with research showing that mental health estimates in refugee populations are generally elevated but vary widely across studies [66]. Our estimate exceeds that of a large-scale study of refugees who arrived in Germany between 2013 and 2016, which reported a prevalence of 41.2% for emotional distress using the same screening instrument [64]. This difference may reflect our sample consisting solely of refugees housed in camps, whereas the comparison study included participants from both private and collective refugee housing and identified refugee housing as a risk factor for increased distress (RR = 1.21). Our estimate also exceeds that from a study conducted at a refugee reception center in Eastern Germany, which reported a 49.7% prevalence of positive screening for somatization, depression, or PTSD using disorder-specific screening instruments [67]. Although these estimates are not directly comparable because different instruments and thresholds were used, both studies indicate a substantial mental health burden in refugee populations.

At the international level, our estimate also exceeds the pooled prevalence of common mental disorders among adult Syrian refugees in high-income Western countries (33%) [68]. However, only studies using diagnostic screening instruments were included in this meta-analysis. Differences in measurement and study quality may partly explain this variation. Furthermore, our study uses a broader definition of refugees, including asylum seekers and individuals in refugee-like situations, including different types of residence status. Hence, it is important to recognize that refugees are a heterogeneous group with diverse sociodemographic backgrounds, which further contributes to the broad range of prevalence estimates across studies.

#### Housing as a determinant of emotional distress

Our findings provide quantitative evidence on housing as a social determinant of mental health in refugee camps. Many housing quality indicators—such as crowding, room size, transfers, safety, lighting, hygiene, autonomy, and frequent feelings of boredom or imprisonment—were associated with emotional distress when examined individually while adjusting for relevant sociodemographic variables. However, when multiple indicators were included in the same regression model, association patterns changed.

Contrary to previous studies linking frequent relocations and forced moves with mental health conditions [69–72], we did not observe a consistent association. Frequent transfers were associated with distress in the single-indicator model, but not in the main model. This may reflect the fact that we did not specifically collect data on whether transfers were forced. Additionally, unlike a longitudinal study measuring the annual relocation rate in asylum-seeking children [69], we counted reported transfers without accounting for the period over which they occurred; three transfers within six months, for example, likely represent a greater disruption than three over five years, so our measure may have attenuated associations.

Safety emerged as a key dimension. Feeling unsafe in one’s own room was associated with increased distress, which is consistent with quantitative, qualitative, and review evidence identifying safety, including the ability to lock one’s door, as a housing quality dimension relevant to the mental health in refugee populations [18, 31, 34]. Lighting conditions were also consistently associated with distress, showing that basic physical housing conditions were related to emotional distress in our sample.

Institutional dimensions of the refugee camp showed some of the most consistent associations with emotional distress. Greater restrictions on autonomy were associated with higher emotional distress. Although conducted in a different population and with the direction of association modeled differently, a cross-sectional Dutch study of rehabilitation patients from five diagnostic groups likewise found a close association between emotional distress and restrictions in participation and autonomy [73]. Emotional distress was the most important factor contributing to participation restrictions in that study. Furthermore, higher frequencies of feeling bored or imprisoned were consistently associated with higher emotional distress in our analyses.

Among sociodemographic variables, female gender and Afghan origin were consistently associated with higher distress. Contrary to evidence from Greece linking length of stay in camps with an acute mental health crisis [74], we observed no association between length of stay in Germany and emotional distress. Although the studies measured different periods of residence (time in a camp versus time in Germany), this difference alone may not explain the contrasting findings. More importantly, the Moria study collected data in 2018 under severely overcrowded conditions, whereas the camps in our study were not similarly overcrowded when we collected data in 2025. These differences in camp conditions may have contributed to the contrasting findings. Being married and dependency on interpreters were not consistently associated with distress across sensitivity analyses. As 63% of married participants reported not living with their spouse in Germany, and family separation and concern for family members have been shown to be associated with poorer mental health among refugees [75, 76], marital status alone may not adequately capture family circumstances. Self-rated health status was among the strongest and most consistent predictors of emotional distress. This finding is consistent with previous evidence linking poor subjective health status to mental health conditions [65].

Taken together, these findings support a multidimensional understanding of housing as a determinant of mental health. Several dimensions, particularly safety, lighting conditions, and institutional practices, remained independently associated with distress when entered simultaneously. Room size was also associated with distress in the primary model, although this association was less robust in the HC3 analysis. These findings suggest partly independent contributions of different housing dimensions rather than a single dominant indicator. Interactions between dimensions were not tested and should be examined in future research. These results have implications for policy and practice. Efforts to improve mental health among refugees should extend beyond improvements to the dwelling’s physical infrastructure, such as building standards and sanitary facilities, and additionally address psychosocial and institutional aspects of refugee housing. Ensuring safe living environments and strengthening residents’ autonomy in refugee camps may be important targets for policy and future intervention research. Future research should develop standardized tools for refugee populations that assess context-specific aspects of housing quality. Such knowledge is essential for adequate quantitative analysis of housing as a social determinant of health.

### Strengths and limitations

A major strength of this study is the multilingual and multidisciplinary composition of our study team. Native-language proficiency and migration backgrounds among several members may have facilitated trust and communication during fieldwork. Recruitment literature on hard-to-reach populations describes the value of community-based recruiters with privileged access to target populations [77], which was realized in this study through the composition of the study team.

Providing the questionnaire in the most common native languages of residents in participating camps, combined with the option of self-completion or interview-administered completion by the study team, enabled inclusion of participants with varying literacy levels and language proficiency. The availability of mobile devices and internet access further reduced access-related barriers. Additionally, the study offers a comprehensive assessment of housing quality, encompassing physical, institutional, and psychosocial dimensions relevant to refugee camp settings and accounting for relevant sociodemographic variables rather than focusing on dichotomized classifications or singular aspects.

At the same time, several limitations should be considered. First, the cross-sectional design does not allow causal inferences; reverse causation is also possible, as individuals with high emotional distress may perceive their environment more negatively. This applies in particular to boredom, which may partly overlap with loss of interest. Second, the study population was restricted to refugees living in camps in three federal states in Eastern Germany. Findings may not be generalizable to those living in private housing, who may be more integrated into German society and face different stressors, or, given our predominantly male sample, mostly of Syrian and Afghan origin, and the language-based eligibility criteria, to refugee populations more broadly. As no population data on refugees living in camps in Eastern Germany were available, representativeness could not be assessed, and weighting could not be applied. Third, the sampling frame was incomplete because not all district authorities provided camp lists, and excluding smaller camps likely reduced external validity. Staff involvement in the initial contact may have influenced who was approached or invited to communal rooms, and residents absent during the visits may be underrepresented despite visits at different times of day and the option of later participation.

Fourth, neither non-participation nor the mode of completion was systematically documented, limiting assessment of selection bias and mode effects. Fifth, as no identifying information such as IP addresses was stored and the survey remained accessible after the visits, repeated participation could not be excluded. Sixth, potentially relevant variables—including residence status, educational background, noise rating, and length of residence in the camp—were mistakenly omitted from the final version of the questionnaire and therefore not assessed. Seventh, adopting crowding response options from public transportation research may not fully capture residential crowding constructs, thereby reducing content validity. Eighth, the Kurdish (Kurmanji) and Turkish versions of the RHS-13 have not been cross-culturally validated. Ninth, attrition was considerable, and missingness not at random cannot be ruled out. Tenth, although the main model had approximately 15.5 observations per predictor coefficient, exceeding the approximately two subjects per variable found sufficient for accurate coefficient estimation under the specific simulation conditions examined by Austin and Steyerberg [78], this comparison does not establish adequate statistical power for all associations; the findings should therefore not be interpreted as confirmatory. Additionally, given the exploratory nature of this study, no a priori sample size calculation was performed. Finally, housing satisfaction was not measured as a separate variable. Our outcome captures emotional symptom burden rather than global well-being.

## Conclusions

This study found that perceived housing quality was closely associated with emotional distress among refugees housed in Eastern German camps. Psychosocial and institutional dimensions of housing quality were particularly relevant to emotional distress. Beyond structural housing conditions, perceived autonomy, safety, and feeling imprisoned emerged as relevant correlates of emotional distress. These findings highlight the need for a broader understanding of housing quality that extends beyond infrastructural standards to encompass experiential aspects of living in refugee camps. Addressing psychosocial housing-related stressors may complement individual-level mental health services and could contribute to improving mental health conditions in this population.

## Supplementary Information

Supplementary Material 1.

Supplementary Material 2.

Supplementary Material 3.

Supplementary Material 4.

## Data Availability

The datasets generated and analyzed during the current study are not publicly available due to data protection and ethical restrictions. Data may only be made available to third parties following clarification with and, where required, approval by the responsible ethics committee. Requests may be directed to the corresponding author.

## Abbreviations

95% CI: 95% Confidence Interval
EU: European Union
FMI: Fraction of Missing Information
HC3: Heteroscedasticity-Consistent Variance Estimator, Type 3
HHGL: WHO Housing and Health Guidelines
IPAQ: Impact on Participation and Autonomy Questionnaire
MAR: Missing at Random
PTSD: Post-Traumatic Stress Disorder
Ref.: Reference Category
RHS-13: Refugee Health Screener-13
RHS-15: Refugee Health Screener-15
RR: Relative Risk
SF-12: Short-Form Health Survey
SHED: Small-area Housing Environment Deterioration
UN: United Nations
WHO: World Health Organization
