# Supplementary material for: Perceived housing quality and emotional distress among refugees living in camps: a cross-sectional study in Eastern Germany
Source: BMC Public Health. 2026 Nov 14;26:3381. doi: 10.1186/s12889-026-30396-8 (PMC13770332; doi:10.1186/s12889-026-30396-8)
Supplement: Supplementary file 1 [file 12889_2026_30396_MOESM1_ESM.docx]

## Questionnaire items

| Indicator | Item code and text | Response code and text | Source and adaptation | Original language and translations | Missing data  *n* (%) |
| --- | --- | --- | --- | --- | --- |
| Place of current residence | F1 Where are you currently staying? | Single choice from a list of the visited camps | o.i. | Original: de; New: ar, en, fa, fr, kmr, ps, tr | 50 (12.2) |
| Residential constellation in own room | F2 How are you currently accommodated in your room? | (1) I am staying alone in the room (2) I am staying with family in the room (3) I am staying with friends or acquaintances in the room (4) I am staying with other residents in the room | o.i. | Original: de; New: ar, en, fa, fr, kmr, ps, tr | 20 (4.9) |
| Possibility to store personal items in own room | F3 Do you have lockable cabinets or compartments for storing personal items in your room? | (1) Yes, (2) No | o.i. | Original: de; New: ar, en, fa, fr, kmr, ps, tr | 27 (6.6) |
| Room inspections | F4 Are there room inspections by staff in your accommodation? | (1) Yes, (2) No | o.i. | Original: de; New: ar, en, fa, fr, kmr, ps, tr | 34 (8.3) |
| Possibility to lock own room | F5 Can you lock your room? | (1) Yes, (2) No | o.i. | Original: de; New: ar, en, fa, fr, kmr, ps, tr | 26 (6.3) |
|  | How are you provided with food in the accommodation? |  | o.i. | Original: de; New: ar, en, fa, fr, kmr, ps, tr |  |
| Availability of own kitchen | F6.SQ001. I have my own kitchen in the accommodation | (1) Yes, (2) No |  |  | 0 (0.0) |
| Sharing a kitchen with other residents | F6.SQ002. I share a kitchen with other residents in the accommodation | (1) Yes, (2) No |  |  | 0 (0.0) |
| Central food provision (e.g. via daily catering) | F6.SQ003. I get food from the accommodation every day (e.g. in a cafeteria) | (1) Yes, (2) No |  |  | 0 (0.0) |
| No provision with food at accommodation | F6.SQ004. I am not provided with food in the accommodation | (1) Yes, (2) No |  |  | 0 (0.0) |
| Availability of showers in the camp | F7 Do you have a shower in your accommodation that you are allowed to use? | (1) I have my own shower in the accommodation (2) I share a shower with only same-sex residents in the accommodation (3) I share a shower with residents of different sexes in the accommodation (4) I don't have a shower in the accommodation | o.i. | Original: de; New: ar, en, fa, fr, kmr, ps, tr | 26 (6.3) |
| Availability of toilets in the camp | F8 Do you have a toilet in your accommodation that you are allowed to use? | (1) I have my own toilet in the accommodation (2) I share a toilet with only same-sex residents in the accommodation (3) I share a toilet with residents of different sexes in the accommodation (4) I don't have a toilet in the accommodation | o.i. | Original: de; New: ar, en, fa, fr, kmr, ps, tr | 23 (5.6) |
|  | How do you rate the following aspects of your accommodation? | (1) Very good, (2) Fairly good, (3) Rather bad, (4) Very bad | o.i. | Original: de; New: ar, en, fa, fr, kmr, ps, tr |  |
| Room size | F9.SQ001. Size of your room |  |  |  | 48 (11.7) |
| Lighting conditions (room) | F9.SQ002. Lighting conditions in your room |  |  |  | 53 (12.9) |
| Hot water supply (camp) | F9.SQ003. Hot water supply in the accommodation |  |  |  | 53 (12.9) |
| Hygiene (shower) | F9.SQ004. Hygiene conditions of the shower |  |  |  | 29 (7.1) |
| Hygiene (toilet) | F9.SQ005. Hygiene conditions of the toilet |  |  |  | 33 (8.0) |
| Hygiene (kitchen) | F9.SQ006. Hygiene conditions of the kitchen |  |  |  | 29 (7.1) |
| Food quality | F9.SQ007. Quality of the food served |  |  |  | 2 (0.5) |
| Food quantity | F9.SQ008. Quantity of the food served |  |  |  | 3 (0.7) |
|  | How do you rate the number of people in the following areas in relation to the available space at your accommodation? | (1) Extremely intolerable, (2) Intolerable, (3) Tolerable, (4) Adequate | Li, 2013: the text “rate the number of people in the following areas in relation to the available space” instead of “describe the level of crowding” and Response option “4= No crowding at all” was replaced by “Adequate” | Original: en; New: ar, fa, fr, kmr, ps, tr |  |
| Crowding (camp) | F10.SQ001. Number of people staying with you in the accommodation |  |  |  | 55 (13.4) |
| Crowding (room) | F10.SQ002. Number of people staying with you in the room |  |  |  | 29 (7.1) |
| Crowding (kitchen) | F10.SQ003. Number of people you share a kitchen with |  |  |  | 25 (6.1) |
| Crowding (shower) | F10.SQ004. Number of people you share a shower with |  |  |  | 24 (5.9) |
| Crowding (toilet) | F10.SQ005. Number of people you share a toilet with |  |  |  | 26 (6.3) |
|  | How safe do you personally feel in the following areas in the accommodation? | (1) Very safe, (2) Rather safe, (3) Rather Unsafe, (4) Very Unsafe | o.i. | Original: de; New: ar, en, fa, fr, kmr, ps, tr |  |
| Safety (camp) | F11.SQ001. In the accommodation |  |  |  | 55 (13.4) |
| Safety (room) | F11.SQ002. In your room |  |  |  | 55 (13.4) |
| Safety (shower) | F11.SQ003. Using the shower |  |  |  | 31 (7.6) |
| Safety (toilet) | F11.SQ004. Using the toilet |  |  |  | 31 (7.6) |
| Autonomy regarding: | How do you rate your ability to need the following needs in the accommodation facility? | (1) Very good, (2) Good, (3) Fair, (4) Bad, (5) Very bad |  | Original: en; New: ar, fa, fr, kmr, ps, tr |  |
| eating and drinking | F12.SQ001. My chances of eating and drinking when I want to are |  | IPAQ, item 2e |  | 54 (13.2) |
| getting up and going to bed | F12.SQ002. My chances of getting up and going to bed when I want to are |  | IPAQ, item 2c |  | 56 (13.7) |
| doing the laundry | F12.SQ003. My chances of doing the laundry when I wish and need to are |  | o.i. |  | 56 (13.7) |
| using the shower | F12.SQ004. My chances of using the shower when I wish and need to are |  | IPAQ, item 2b: “getting washed and dressed when I want to” was replaced by “using the shower when I wish and need to” |  | 54 (13.2) |
| using the toilet | F12.SQ005. My chances of going to the toilet when I wish and need to are |  | IPAQ, item 2d |  | 57 (13.9) |
| looking after own room | F12.SQ006. My chances of contributing to looking after my room the way I want to are |  | IPAQ,item 3a: “home” was replaced by “room” |  | 58 (14.1) |
| getting repairs done | F12.SQ007. My chances of getting minor repairs and maintenance work done in my room when I wish and need to are |  | IPAQ; item 3e; Adapted: “house and garden” was replaced by “room” and “by myself or by others” was deleted |  | 57 (13.9) |
| retreating to a private area | F12.SQ008. My chances of being able to retreat to a private area when I want to are |  | o.i. |  | 59 (14.4) |
| practicing personal activities | F12.SQ009. My chances of practicing my religious or cultural traditions (e.g. praying, fasting) undisturbed when I want to are |  | o.i. |  | 60 (14.6) |
| Frequency of feeling bored | F13 How often or how rarely have you felt bored in the accommodation facility in the past two weeks? | (1) Never, (2) Rarely, (3) Sometimes, (4) Often, (5) Always | o.i. | Original: de; New: ar, en, fa, fr, kmr, ps, tr | 59 (14.4) |
| Frequency of feeling imprisoned | F14 How often or how rarely have you felt like you were in prison in the accommodation facility in the past two weeks? | (1) Never, (2) Rarely, (3) Sometimes, (4) Often, (5) Always | o.i. | Original: de; New: ar, en, fa, fr, kmr, ps, tr | 62 (15.1) |
| Emotional distress | F17.SQ001. | (0) Not at all, (1) A little bit, (2) Moderately, (3) Quite a lot, (4) Extremely | RHS-13 | Original: en; Existing: ar, fa, fr, ps  New: kmr, tr | 66 (16.1) |
|  | F17.SQ002. |  |  |  | 67 (16.3) |
|  | F17.SQ003. |  |  |  | 67 (16.3) |
|  | F17.SQ004. |  |  |  | 68 (16.6) |
|  | F17.SQ005. |  |  |  | 68 (16.6) |
|  | F17.SQ006. |  |  |  | 68 (16.6) |
|  | F17.SQ007. |  |  |  | 67 (16.3) |
|  | F17.SQ008. |  |  |  | 67 (16.3) |
|  | F17.SQ009. |  |  |  | 68 (16.6) |
|  | F18.SQ001. |  |  |  | 66 (16.1) |
|  | F18.SQ002. |  |  |  | 66 (16.1) |
|  | F18.SQ003. |  |  |  | 66 (16.1) |
|  | F18.SQ004. |  |  |  | 67 (16.3) |
| Subjective general health status | F24 How would you describe your health in general? | (1) Excellent, (2) Very good, (3) Good, (4) Fair, (5) Poor | SF-12 | Original: en; New: ar, fa, fr, kmr, ps, tr | 76 (18.5) |
| Age (years) | F39 How old are you (Please enter your age in years) | Open question | o.i. | Original: de; New: ar, en, fa, fr, kmr, ps, tr | 92 (22.4) |
| Country of origin | F40 Which country were you born in? | (1) Afghanistan, (2) Syria, (3) Iraq, (4) Palestine, (5) Turkey, (6) Iran, (7) India, (8) Ghana, (9) Benin, (10) Nigeria, (11) Burkina Faso, (12) Cameroon, (13) Namibia, (14) Mali, (15) Somalia, Other | o.i. | Original: de; New: ar, en, fa, fr, kmr, ps, tr | 92 (22.4) |
| Gender | F41 Which gender do you feel you belong to? | (1) Male, (2) Female, (3) Diverse | o.i. | Original: de; New: ar, en, fa, fr, kmr, ps, tr | 91 (22.2) |
| Length of stay in Germany (months) | F42 How long have you been living in Germany (Please enter the duration in months) | Open question | o.i. | Original: de; New: ar, en, fa, fr, kmr, ps, tr | 98 (23.9) |
| Number of transfers between camps | F43 How often have you had to change accommodations since arriving in Germany? | (1) 0, (2) 1, (3) 2, (4) 3, (5) 4, (6) 5, (7) > 5 | o.i. | Original: de; New: ar, en, fa, fr, kmr, ps, tr | 94 (22.9) |
| Dependency on language interpreters | F44 Are you dependent on language mediators in your everyday life (e.g. during official visits or doctor's appointments)? | (1) Yes, (2) No | o.i. | Original: de; New: ar, en, fa, fr, kmr, ps, tr | 91 (22.2) |
| Religious affiliation | F46 What religion do you belong to? | (1) Christianity, (2) Islam, (3) Hinduism, (4) Buddhism, (5) Judaism, (6) None, Other | o.i. | Original: de; New: ar, en, fa, fr, kmr, ps, tr | 109 (26.6) |
| Current marital status | F47 What is your current family status? | (1) Married, (2) Single, (3) Divorced, (4) Widowed | o.i. | Original: de; New: ar, en, fa, fr, kmr, ps, tr | 90 (22.0) |
| Separation from spouse in Germany | F47a Is your spouse also living in Germany? | (1) Yes, (2) No | o.i. | Original: de; New: ar, en, fa, fr, kmr, ps, tr | 90 (22.0) |
| Separation from spouse in the camp | F47b Do you live together in the same accommodation? | (1) Yes, (2) No | o.i. | Original: de; New: ar, en, fa, fr, kmr, ps, tr | 90 (22.0) |
| Children | F48 Do you have children? | (1) Yes, (2) No | o.i. | Original: de; New: ar, en, fa, fr, kmr, ps, tr | 91 (22.2) |
| Separation from children in Germany | F48a Are your children also living in Germany? | (1) All my children are living in Germany (2) Not all my children are living in Germany (3) All my children are not living in Germany | o.i. | Original: de; New: ar, en, fa, fr, kmr, ps, tr | 91 (22.2) |
| Separation from children in the camp | F48b Do you live together in the same accommodation? | (1) I live with all the children in the same accommodation facility (2) I live with some but not all of the children in the same accommodation facility (3) I do not live with any of my children in the same accommodation facility | o.i. | Original: de; New: ar, en, fa, fr, kmr, ps, tr | 0 (0.0) |
