# Supplementary material for: Perceived housing quality and emotional distress among refugees living in camps: a cross-sectional study in Eastern Germany
Source: BMC Public Health. 2026 Nov 14;26:3381. doi: 10.1186/s12889-026-30396-8 (PMC13770332; doi:10.1186/s12889-026-30396-8)
Supplement: Supplementary file 2 [file 12889_2026_30396_MOESM2_ESM.docx]

## Descriptive statistics of original (non-combined) categories for all housing quality indicators

| **Variable** | **Available cases** | **Overall  % (n)  [95% CI]** | **Available cases** | **Negative screening**  **(N = 72)  % (n)  [95% CI]** | **Mild distress**  **(N = 56)  % (n)  [95% CI]** | **Moderate distress**  **(N = 51)  % (n)  [95% CI]** | **Severe distress**  **(N = 164)  % (n)  [95% CI]** | **Total screening**  **(N = 343)  % (n)  [95% CI]** |
| --- | --- | --- | --- | --- | --- | --- | --- | --- |
| ***Crowding (room)*** | 291 |  | 273 |  |  |  |  |  |
| *Adequate* |  | 26.1 (76)  [21, 32] |  | 50.0 (28)  [37, 63] | 30.4 (14)  [18, 46] | 17.5 (7)  [7.9, 33] | 16.8 (22)  [11, 25] | 26.0 (71)  [21, 32] |
| *Tolerable* |  | 29.2 (85)  [24, 35] |  | 30.4 (17)  [19, 44] | 28.3 (13)  [16, 44] | 25.0 (10)  [13, 42] | 30.5 (40)  [23, 39] | 29.3 (80)  [24, 35] |
| *Intolerable* |  | 26.8 (78)  [22, 32] |  | 14.3 (8)  [6.8, 27] | 34.8 (16)  [22, 50] | 30.0 (12)  [17, 47] | 29.8 (39)  [22, 38] | 27.5 (75)  [22, 33] |
| *Extremely intolerable* |  | 17.9 (52)  [14, 23] |  | 5.4 (3)  [1.4, 16] | 6.5 (3)  [1.7, 19] | 27.5 (11)  [15, 44] | 22.9 (30)  [16, 31] | 17.2 (47)  [13, 22] |
| ***Crowding (accommodation)*** | 355 |  | 334 |  |  |  |  |  |
| *Adequate* |  | 25.6 (91)  [21, 31] |  | 44.9 (31)  [33, 57] | 26.8 (15)  [16, 41] | 23.4 (11)  [13, 38] | 17.3 (28)  [12, 24] | 25.4 (85)  [21, 31] |
| *Tolerable* |  | 38.6 (137)  [34, 44] |  | 39.1 (27)  [28, 52] | 48.2 (27)  [35, 62] | 40.4 (19)  [27, 56] | 35.2 (57)  [28, 43] | 38.9 (130)  [34, 44] |
| *Intolerable* |  | 22.3 (79)  [18, 27] |  | 13.0 (9)  [6.5, 24] | 19.6 (11)  [11, 33] | 23.4 (11)  [13, 38] | 27.8 (45)  [21, 35] | 22.8 (76)  [18, 28] |
| *Extremely intolerable* |  | 13.5 (48)  [10, 18] |  | 2.9 (2)  [0.50, 11] | 5.4 (3)  [1.4, 16] | 12.8 (6)  [5.3, 26] | 19.8 (32)  [14, 27] | 12.9 (43)  [9.6, 17] |
| ***Crowding (kitchen)*** | 298 |  | 283 |  |  |  |  |  |
| *Adequate* |  | 18.8 (56)  [15, 24] |  | 44.6 (25)  [32, 58] | 17.0 (8)  [8.1, 31] | 18.6 (8)  [8.9, 34] | 8.0 (11)  [4.3, 14] | 18.4 (52)  [14, 23] |
| *Tolerable* |  | 31.9 (95)  [27, 38] |  | 33.9 (19)  [22, 48] | 38.3 (18)  [25, 54] | 32.6 (14)  [20, 49] | 29.9 (41)  [23, 38] | 32.5 (92)  [27, 38] |
| *Intolerable* |  | 23.2 (69)  [19, 28] |  | 14.3 (8)  [6.8, 27] | 21.3 (10)  [11, 36] | 25.6 (11)  [14, 41] | 27.0 (37)  [20, 35] | 23.3 (66)  [19, 29] |
| *Extremely intolerable* |  | 26.2 (78)  [21, 32] |  | 7.1 (4)  [2.3, 18] | 23.4 (11)  [13, 38] | 23.3 (10)  [12, 39] | 35.0 (48)  [27, 44] | 25.8 (73)  [21, 31] |
| ***Crowding (shower)*** | 279 |  | 267 |  |  |  |  |  |
| *Adequate* |  | 12.5 (35)  [9.0, 17] |  | 30.0 (15)  [18, 45] | 13.3 (6)  [5.5, 27] | 10.3 (4)  [3.3, 25] | 5.3 (7)  [2.3, 11] | 12.0 (32)  [8.5, 17] |
| *Tolerable* |  | 35.1 (98)  [30, 41] |  | 38.0 (19)  [25, 53] | 42.2 (19)  [28, 58] | 35.9 (14)  [22, 53] | 32.3 (43)  [25, 41] | 35.6 (95)  [30, 42] |
| *Intolerable* |  | 25.4 (71)  [21, 31] |  | 22.0 (11)  [12, 36] | 22.2 (10)  [12, 37] | 28.2 (11)  [16, 45] | 28.6 (38)  [21, 37] | 26.2 (70)  [21, 32] |
| *Extremely intolerable* |  | 26.9 (75)  [22, 33] |  | 10.0 (5)  [3.7, 23] | 22.2 (10)  [12, 37] | 25.6 (10)  [14, 42] | 33.8 (45)  [26, 43] | 26.2 (70)  [21, 32] |
| ***Crowding (toilet)*** | 307 |  | 292 |  |  |  |  |  |
| *Adequate* |  | 12.4 (38)  [9.0, 17] |  | 25.9 (15)  [16, 39] | 14.9 (7)  [6.7, 29] | 7.3 (3)  [1.9, 21] | 6.8 (10)  [3.5, 13] | 12.0 (35)  [8.6, 16] |
| *Tolerable* |  | 30.9 (95)  [26, 36] |  | 36.2 (21)  [24, 50] | 31.9 (15)  [20, 47] | 41.5 (17)  [27, 58] | 26.0 (38)  [19, 34] | 31.2 (91)  [26, 37] |
| *Intolerable* |  | 28.0 (86)  [23, 33] |  | 24.1 (14)  [14, 37] | 36.2 (17)  [23, 52] | 24.4 (10)  [13, 41] | 28.1 (41)  [21, 36] | 28.1 (82)  [23, 34] |
| *Extremely intolerable* |  | 28.7 (88)  [24, 34] |  | 13.8 (8)  [6.6, 26] | 17.0 (8)  [8.1, 31] | 26.8 (11)  [15, 43] | 39.0 (57)  [31, 47] | 28.8 (84)  [24, 34] |
| ***Room size*** | 362 |  | 339 |  |  |  |  |  |
| *Very good* |  | 13.5 (49)  [10, 18] |  | 18.3 (13)  [10, 30] | 19.6 (11)  [11, 33] | 8.3 (4)  [2.7, 21] | 9.1 (15)  [5.4, 15] | 12.7 (43)  [9.4, 17] |
| *Fairly good* |  | 43.6 (158)  [38, 49] |  | 60.6 (43)  [48, 72] | 46.4 (26)  [33, 60] | 45.8 (22)  [32, 61] | 35.4 (58)  [28, 43] | 44.0 (149)  [39, 49] |
| *Rather bad* |  | 21.5 (78)  [17, 26] |  | 15.5 (11)  [8.4, 26] | 14.3 (8)  [6.8, 27] | 20.8 (10)  [11, 35] | 26.8 (44)  [20, 34] | 21.5 (73)  [17, 26] |
| *Very bad* |  | 21.3 (77)  [17, 26] |  | 5.6 (4)  [1.8, 15] | 19.6 (11)  [11, 33] | 25.0 (12)  [14, 40] | 28.7 (47)  [22, 36] | 21.8 (74)  [18, 27] |
| ***Availability of own kitchens*** | 410 |  | 343 |  |  |  |  |  |
| *Yes* |  | 8.8 (36)  [6.3, 12] |  | 8.3 (6)  [3.4, 18] | 10.7 (6)  [4.4, 23] | 5.9 (3)  [1.5, 17] | 6.7 (11)  [3.6, 12] | 7.6 (26)  [5.1, 11] |
| *No* |  | 91.2 (374)  [88, 94] |  | 91.7 (66)  [82, 97] | 89.3 (50)  [77, 96] | 94.1 (48)  [83, 98] | 93.3 (153)  [88, 96] | 92.4 (317)  [89, 95] |
| ***Availability of shared kitchens*** | 410 |  | 343 |  |  |  |  |  |
| *Yes* |  | 78.8 (323)  [74, 83] |  | 81.9 (59)  [71, 90] | 83.9 (47)  [71, 92] | 90.2 (46)  [78, 96] | 84.1 (138)  [77, 89] | 84.5 (290)  [80, 88] |
| *No* |  | 21.2 (87)  [17, 26] |  | 18.1 (13)  [10, 29] | 16.1 (9)  [8.1, 29] | 9.8 (5)  [3.7, 22] | 15.9 (26)  [11, 23] | 15.5 (53)  [12, 20] |
| ***Availability of showers*** | 384 |  | 339 |  |  |  |  |  |
| *Having own shower* |  | 14.6 (56)  [11, 19] |  | 16.7 (12)  [9.3, 28] | 16.1 (9)  [8.1, 29] | 12.0 (6)  [5.0, 25] | 10.6 (17)  [6.5, 17] | 13.0 (44)  [9.7, 17] |
| *Sharing shower with only same-sex residents* |  | 69.5 (267)  [65, 74] |  | 63.9 (46)  [52, 75] | 73.2 (41)  [59, 84] | 78.0 (39)  [64, 88] | 72.0 (116)  [64, 79] | 71.4 (242)  [66, 76] |
| *Sharing shower with different-sex residents* |  | 9.4 (36)  [6.7, 13] |  | 9.7 (7)  [4.3, 20] | 7.1 (4)  [2.3, 18] | 4.0 (2)  [0.70, 15] | 11.2 (18)  [6.9, 17] | 9.1 (31)  [6.4, 13] |
| *No shower in accommodation* |  | 6.5 (25)  [4.3, 9.6] |  | 9.7 (7)  [4.3, 20] | 3.6 (2)  [0.62, 13] | 6.0 (3)  [1.6, 18] | 6.2 (10)  [3.2, 11] | 6.5 (22)  [4.2, 9.8] |
| ***Availability of toilets*** | 387 |  | 341 |  |  |  |  |  |
| *Having own toilet* |  | 13.4 (52)  [10, 17] |  | 16.7 (12)  [9.3, 28] | 16.1 (9)  [8.1, 29] | 12.0 (6)  [5.0, 25] | 8.6 (14)  [5.0, 14] | 12.0 (41)  [8.9, 16] |
| *Sharing toilet with only same-sex residents* |  | 74.2 (287)  [69, 78] |  | 72.2 (52)  [60, 82] | 75.0 (42)  [61, 85] | 80.0 (40)  [66, 89] | 76.7 (125)  [69, 83] | 76.0 (259)  [71, 80] |
| *Sharing toilet with different-sex residents* |  | 11.9 (46)  [8.9, 16] |  | 11.1 (8)  [5.3, 21] | 8.9 (5)  [3.3, 20] | 6.0 (3)  [1.6, 18] | 14.1 (23)  [9.3, 21] | 11.4 (39)  [8.4, 15] |
| *No toilet in accommodation* |  | 0.5 (2)  [0.09, 2.1] |  | 0.0 (0)  [0.00, 6.3] | 0.0 (0)  [0.00, 8.0] | 2.0 (1)  [0.10, 12] | 0.6 (1)  [0.03, 3.9] | 0.6 (2)  [0.10, 2.3] |
| ***Possibility to lock own rooms*** | 384 |  | 339 |  |  |  |  |  |
| *Yes* |  | 93.5 (359)  [90, 96] |  | 94.4 (67)  [85, 98] | 96.4 (53)  [86, 99] | 96.0 (48)  [85, 99] | 90.8 (148)  [85, 95] | 93.2 (316)  [90, 96] |
| *No* |  | 6.5 (25)  [4.3, 9.6] |  | 5.6 (4)  [1.8, 15] | 3.6 (2)  [0.63, 14] | 4.0 (2)  [0.70, 15] | 9.2 (15)  [5.4, 15] | 6.8 (23)  [4.4, 10] |
| ***Possibility to store personal items (room)*** | 383 |  | 338 |  |  |  |  |  |
| *Yes* |  | 65.5 (251)  [61, 70] |  | 71.8 (51)  [60, 82] | 63.6 (35)  [50, 76] | 67.3 (33)  [52, 80] | 62.0 (101)  [54, 69] | 65.1 (220)  [60, 70] |
| *No* |  | 34.5 (132)  [30, 39] |  | 28.2 (20)  [18, 40] | 36.4 (20)  [24, 50] | 32.7 (16)  [20, 48] | 38.0 (62)  [31, 46] | 34.9 (118)  [30, 40] |
| ***Accommodation transfers*** | 316 |  | 316 |  |  |  |  |  |
| *No transfers* |  | 12.0 (38)  [8.8, 16] |  | 23.9 (16)  [15, 36] | 10.0 (5)  [3.7, 23] | 6.1 (3)  [1.6, 18] | 9.3 (14)  [5.4, 15] | 12.0 (38)  [8.8, 16] |
| *1–2 transfers* |  | 54.7 (173)  [49, 60] |  | 56.7 (38)  [44, 69] | 56.0 (28)  [41, 70] | 67.3 (33)  [52, 80] | 49.3 (74)  [41, 58] | 54.7 (173)  [49, 60] |
| *≥ 3 transfers* |  | 33.2 (105)  [28, 39] |  | 19.4 (13)  [11, 31] | 34.0 (17)  [22, 49] | 26.5 (13)  [15, 41] | 41.3 (62)  [33, 50] | 33.2 (105)  [28, 39] |
| ***Safety (room)*** | 355 |  | 334 |  |  |  |  |  |
| *Very safe* |  | 38.9 (138)  [34, 44] |  | 62.9 (44)  [50, 74] | 46.4 (26)  [33, 60] | 29.8 (14)  [18, 45] | 27.3 (44)  [21, 35] | 38.3 (128)  [33, 44] |
| *Rather safe* |  | 40.6 (144)  [35, 46] |  | 35.7 (25)  [25, 48] | 41.1 (23)  [28, 55] | 51.1 (24)  [36, 66] | 40.4 (65)  [33, 48] | 41.0 (137)  [36, 47] |
| *Rather unsafe* |  | 11.5 (41)  [8.5, 15] |  | 1.4 (1)  [0.07, 8.8] | 7.1 (4)  [2.3, 18] | 19.1 (9)  [9.6, 34] | 16.1 (26)  [11, 23] | 12.0 (40)  [8.8, 16] |
| *Very unsafe* |  | 9.0 (32)  [6.3, 13] |  | 0.0 (0)  [0.00, 6.5] | 5.4 (3)  [1.4, 16] | 0.0 (0)  [0.00, 9.4] | 16.1 (26)  [11, 23] | 8.7 (29)  [6.0, 12] |
| ***Safety (accommodation)*** | 355 |  | 333 |  |  |  |  |  |
| *Very safe* |  | 34.4 (122)  [29, 40] |  | 52.9 (37)  [41, 65] | 50.0 (28)  [37, 63] | 26.1 (12)  [15, 41] | 21.1 (34)  [15, 28] | 33.3 (111)  [28, 39] |
| *Rather safe* |  | 41.7 (148)  [37, 47] |  | 40.0 (28)  [29, 52] | 42.9 (24)  [30, 57] | 45.7 (21)  [31, 61] | 42.2 (68)  [35, 50] | 42.3 (141)  [37, 48] |
| *Rather unsafe* |  | 12.7 (45)  [9.5, 17] |  | 7.1 (5)  [2.7, 17] | 5.4 (3)  [1.4, 16] | 23.9 (11)  [13, 39] | 15.5 (25)  [10, 22] | 13.2 (44)  [9.9, 17] |
| *Very unsafe* |  | 11.3 (40)  [8.3, 15] |  | 0.0 (0)  [0.00, 6.5] | 1.8 (1)  [0.09, 11] | 4.3 (2)  [0.76, 16] | 21.1 (34)  [15, 28] | 11.1 (37)  [8.0, 15] |
| ***Safety (shower)*** | 328 |  | 309 |  |  |  |  |  |
| *Very safe* |  | 32.6 (107)  [28, 38] |  | 49.2 (31)  [37, 62] | 37.0 (20)  [25, 51] | 29.5 (13)  [17, 45] | 21.6 (32)  [15, 29] | 31.1 (96)  [26, 37] |
| *Rather safe* |  | 39.0 (128)  [34, 45] |  | 44.4 (28)  [32, 57] | 42.6 (23)  [30, 57] | 47.7 (21)  [33, 63] | 34.5 (51)  [27, 43] | 39.8 (123)  [34, 46] |
| *Rather unsafe* |  | 15.9 (52)  [12, 20] |  | 4.8 (3)  [1.2, 14] | 13.0 (7)  [5.8, 26] | 13.6 (6)  [5.7, 28] | 22.3 (33)  [16, 30] | 15.9 (49)  [12, 21] |
| *Very unsafe* |  | 12.5 (41)  [9.2, 17] |  | 1.6 (1)  [0.08, 9.7] | 7.4 (4)  [2.4, 19] | 9.1 (4)  [3.0, 23] | 21.6 (32)  [15, 29] | 13.3 (41)  [9.8, 18] |
| ***Safety (toilet)*** | 354 |  | 333 |  |  |  |  |  |
| *Very safe* |  | 35.0 (124)  [30, 40] |  | 52.9 (37)  [41, 65] | 42.9 (24)  [30, 57] | 30.4 (14)  [18, 46] | 23.6 (38)  [17, 31] | 33.9 (113)  [29, 39] |
| *Rather safe* |  | 38.4 (136)  [33, 44] |  | 42.9 (30)  [31, 55] | 35.7 (20)  [24, 50] | 45.7 (21)  [31, 61] | 36.6 (59)  [29, 45] | 39.0 (130)  [34, 45] |
| *Rather unsafe* |  | 13.6 (48)  [10, 18] |  | 4.3 (3)  [1.1, 13] | 12.5 (7)  [5.6, 25] | 13.0 (6)  [5.4, 27] | 18.0 (29)  [13, 25] | 13.5 (45)  [10, 18] |
| *Very unsafe* |  | 13.0 (46)  [9.8, 17] |  | 0.0 (0)  [0.00, 6.5] | 8.9 (5)  [3.3, 20] | 10.9 (5)  [4.1, 24] | 21.7 (35)  [16, 29] | 13.5 (45)  [10, 18] |
| ***Lighting conditions (room)*** | 357 |  | 336 |  |  |  |  |  |
| *Very good* |  | 30.8 (110)  [26, 36] |  | 40.6 (28)  [29, 53] | 41.1 (23)  [28, 55] | 31.9 (15)  [20, 47] | 22.0 (36)  [16, 29] | 30.4 (102)  [26, 36] |
| *Fairly good* |  | 53.5 (191)  [48, 59] |  | 56.5 (39)  [44, 68] | 50.0 (28)  [37, 63] | 44.7 (21)  [30, 60] | 56.1 (92)  [48, 64] | 53.6 (180)  [48, 59] |
| *Rather bad* |  | 10.9 (39)  [8.0, 15] |  | 1.4 (1)  [0.08, 8.9] | 5.4 (3)  [1.4, 16] | 12.8 (6)  [5.3, 26] | 16.5 (27)  [11, 23] | 11.0 (37)  [8.0, 15] |
| *Very bad* |  | 4.8 (17)  [2.9, 7.7] |  | 1.4 (1)  [0.08, 8.9] | 3.6 (2)  [0.62, 13] | 10.6 (5)  [4.0, 24] | 5.5 (9)  [2.7, 10] | 5.1 (17)  [3.1, 8.1] |
| ***Hot water supply*** | 357 |  | 335 |  |  |  |  |  |
| *Very good* |  | 57.1 (204)  [52, 62] |  | 64.3 (45)  [52, 75] | 58.2 (32)  [44, 71] | 66.7 (32)  [51, 79] | 50.0 (81)  [42, 58] | 56.7 (190)  [51, 62] |
| *Fairly good* |  | 37.0 (132)  [32, 42] |  | 31.4 (22)  [21, 44] | 40.0 (22)  [27, 54] | 25.0 (12)  [14, 40] | 42.0 (68)  [34, 50] | 37.0 (124)  [32, 42] |
| *Rather bad* |  | 4.5 (16)  [2.7, 7.3] |  | 4.3 (3)  [1.1, 13] | 1.8 (1)  [0.09, 11] | 4.2 (2)  [0.72, 15] | 6.2 (10)  [3.2, 11] | 4.8 (16)  [2.8, 7.8] |
| *Very bad* |  | 1.4 (5)  [0.52, 3.4] |  | 0.0 (0)  [0.00, 6.5] | 0.0 (0)  [0.00, 8.1] | 4.2 (2)  [0.72, 15] | 1.9 (3)  [0.48, 5.7] | 1.5 (5)  [0.55, 3.6] |
| ***Hygiene (kitchen)*** | 321 |  | 301 |  |  |  |  |  |
| *Very good* |  | 20.2 (65)  [16, 25] |  | 37.1 (23)  [25, 50] | 23.5 (12)  [13, 38] | 14.0 (6)  [5.8, 29] | 13.1 (19)  [8.3, 20] | 19.9 (60)  [16, 25] |
| *Fairly good* |  | 39.9 (128)  [35, 45] |  | 50.0 (31)  [38, 62] | 47.1 (24)  [33, 61] | 37.2 (16)  [23, 53] | 35.2 (51)  [28, 44] | 40.5 (122)  [35, 46] |
| *Rather bad* |  | 20.2 (65)  [16, 25] |  | 8.1 (5)  [3.0, 19] | 17.6 (9)  [8.9, 31] | 23.3 (10)  [12, 39] | 24.1 (35)  [18, 32] | 19.6 (59)  [15, 25] |
| *Very bad* |  | 19.6 (63)  [16, 24] |  | 4.8 (3)  [1.3, 14] | 11.8 (6)  [4.9, 25] | 25.6 (11)  [14, 41] | 27.6 (40)  [21, 36] | 19.9 (60)  [16, 25] |
| ***Hygiene (shower)*** | 330 |  | 310 |  |  |  |  |  |
| *Very good* |  | 24.5 (81)  [20, 30] |  | 34.4 (22)  [23, 47] | 25.9 (14)  [15, 40] | 23.3 (10)  [12, 39] | 18.1 (27)  [12, 25] | 23.5 (73)  [19, 29] |
| *Fairly good* |  | 37.0 (122)  [32, 42] |  | 45.3 (29)  [33, 58] | 48.1 (26)  [35, 62] | 32.6 (14)  [20, 49] | 32.9 (49)  [26, 41] | 38.1 (118)  [33, 44] |
| *Rather bad* |  | 16.7 (55)  [13, 21] |  | 7.8 (5)  [2.9, 18] | 14.8 (8)  [7.1, 28] | 16.3 (7)  [7.3, 31] | 20.1 (30)  [14, 28] | 16.1 (50)  [12, 21] |
| *Very bad* |  | 21.8 (72)  [18, 27] |  | 12.5 (8)  [5.9, 24] | 11.1 (6)  [4.6, 23] | 27.9 (12)  [16, 44] | 28.9 (43)  [22, 37] | 22.3 (69)  [18, 27] |
| ***Hygiene (toilet)*** | 352 |  | 332 |  |  |  |  |  |
| *Very good* |  | 21.0 (74)  [17, 26] |  | 27.5 (19)  [18, 40] | 28.6 (16)  [18, 42] | 19.6 (9)  [9.9, 34] | 14.9 (24)  [10, 22] | 20.5 (68)  [16, 25] |
| *Fairly good* |  | 31.8 (112)  [27, 37] |  | 46.4 (32)  [34, 59] | 39.3 (22)  [27, 53] | 30.4 (14)  [18, 46] | 23.6 (38)  [17, 31] | 31.9 (106)  [27, 37] |
| *Rather bad* |  | 16.8 (59)  [13, 21] |  | 8.7 (6)  [3.6, 19] | 21.4 (12)  [12, 35] | 17.4 (8)  [8.3, 32] | 17.4 (28)  [12, 24] | 16.3 (54)  [13, 21] |
| *Very bad* |  | 30.4 (107)  [26, 36] |  | 17.4 (12)  [9.7, 29] | 10.7 (6)  [4.4, 23] | 32.6 (15)  [20, 48] | 44.1 (71)  [36, 52] | 31.3 (104)  [26, 37] |
| ***Region*** | 360 |  | 323 |  |  |  |  |  |
| *Urban* |  | 45.0 (162)  [40, 50] |  | 43.7 (31)  [32, 56] | 54.0 (27)  [39, 68] | 46.9 (23)  [33, 62] | 40.5 (62)  [33, 49] | 44.3 (143)  [39, 50] |
| *Rural* |  | 55.0 (198)  [50, 60] |  | 56.3 (40)  [44, 68] | 46.0 (23)  [32, 61] | 53.1 (26)  [38, 67] | 59.5 (91)  [51, 67] | 55.7 (180)  [50, 61] |
| ***Autonomy regarding eating and drinking*** | 356 |  | 334 |  |  |  |  |  |
| *Very good* |  | 32.6 (116)  [28, 38] |  | 47.8 (33)  [36, 60] | 44.6 (25)  [32, 58] | 34.0 (16)  [21, 49] | 24.1 (39)  [18, 32] | 33.8 (113)  [29, 39] |
| *Good* |  | 28.7 (102)  [24, 34] |  | 31.9 (22)  [21, 44] | 28.6 (16)  [18, 42] | 27.7 (13)  [16, 43] | 27.2 (44)  [21, 35] | 28.4 (95)  [24, 34] |
| *Fair* |  | 24.2 (86)  [20, 29] |  | 14.5 (10)  [7.5, 26] | 17.9 (10)  [9.3, 31] | 31.9 (15)  [20, 47] | 26.5 (43)  [20, 34] | 23.4 (78)  [19, 28] |
| *Poor* |  | 9.0 (32)  [6.3, 13] |  | 5.8 (4)  [1.9, 15] | 5.4 (3)  [1.4, 16] | 6.4 (3)  [1.7, 19] | 12.3 (20)  [7.9, 19] | 9.0 (30)  [6.2, 13] |
| *Very poor* |  | 5.6 (20)  [3.6, 8.7] |  | 0.0 (0)  [0.00, 6.6] | 3.6 (2)  [0.62, 13] | 0.0 (0)  [0.00, 9.4] | 9.9 (16)  [5.9, 16] | 5.4 (18)  [3.3, 8.5] |
| ***Autonomy regarding getting up and going to bed*** | 354 |  | 333 |  |  |  |  |  |
| *Very good* |  | 34.5 (122)  [30, 40] |  | 50.7 (35)  [39, 63] | 45.5 (25)  [32, 59] | 31.9 (15)  [20, 47] | 26.5 (43)  [20, 34] | 35.4 (118)  [30, 41] |
| *Good* |  | 28.8 (102)  [24, 34] |  | 36.2 (25)  [25, 49] | 20.0 (11)  [11, 33] | 36.2 (17)  [23, 52] | 27.2 (44)  [21, 35] | 29.1 (97)  [24, 34] |
| *Fair* |  | 16.9 (60)  [13, 21] |  | 8.7 (6)  [3.6, 19] | 21.8 (12)  [12, 35] | 14.9 (7)  [6.7, 29] | 18.5 (30)  [13, 26] | 16.5 (55)  [13, 21] |
| *Poor* |  | 12.4 (44)  [9.3, 16] |  | 2.9 (2)  [0.50, 11] | 10.9 (6)  [4.5, 23] | 12.8 (6)  [5.3, 26] | 16.7 (27)  [11, 24] | 12.3 (41)  [9.1, 16] |
| *Very poor* |  | 7.3 (26)  [4.9, 11] |  | 1.4 (1)  [0.08, 8.9] | 1.8 (1)  [0.09, 11] | 4.3 (2)  [0.74, 16] | 11.1 (18)  [6.9, 17] | 6.6 (22)  [4.3, 10] |
| ***Autonomy regarding doing the laundry*** | 354 |  | 333 |  |  |  |  |  |
| *Very good* |  | 24.0 (85)  [20, 29] |  | 44.9 (31)  [33, 57] | 28.6 (16)  [18, 42] | 25.5 (12)  [14, 41] | 13.7 (22)  [8.9, 20] | 24.3 (81)  [20, 29] |
| *Good* |  | 26.8 (95)  [22, 32] |  | 31.9 (22)  [21, 44] | 30.4 (17)  [19, 44] | 21.3 (10)  [11, 36] | 26.7 (43)  [20, 34] | 27.6 (92)  [23, 33] |
| *Fair* |  | 22.6 (80)  [18, 27] |  | 18.8 (13)  [11, 30] | 25.0 (14)  [15, 39] | 21.3 (10)  [11, 36] | 23.0 (37)  [17, 30] | 22.2 (74)  [18, 27] |
| *Poor* |  | 16.4 (58)  [13, 21] |  | 2.9 (2)  [0.50, 11] | 10.7 (6)  [4.4, 23] | 25.5 (12)  [14, 41] | 21.1 (34)  [15, 28] | 16.2 (54)  [13, 21] |
| *Very poor* |  | 10.2 (36)  [7.3, 14] |  | 1.4 (1)  [0.08, 8.9] | 5.4 (3)  [1.4, 16] | 6.4 (3)  [1.7, 19] | 15.5 (25)  [10, 22] | 9.6 (32)  [6.8, 13] |
| ***Autonomy regarding using the shower*** | 356 |  | 335 |  |  |  |  |  |
| *Very good* |  | 32.9 (117)  [28, 38] |  | 46.4 (32)  [34, 59] | 42.9 (24)  [30, 57] | 34.0 (16)  [21, 49] | 23.9 (39)  [18, 31] | 33.1 (111)  [28, 38] |
| *Good* |  | 32.0 (114)  [27, 37] |  | 27.5 (19)  [18, 40] | 37.5 (21)  [25, 51] | 38.3 (18)  [25, 54] | 31.9 (52)  [25, 40] | 32.8 (110)  [28, 38] |
| *Fair* |  | 24.2 (86)  [20, 29] |  | 21.7 (15)  [13, 34] | 16.1 (9)  [8.1, 29] | 21.3 (10)  [11, 36] | 25.2 (41)  [19, 33] | 22.4 (75)  [18, 27] |
| *Poor* |  | 9.0 (32)  [6.3, 13] |  | 4.3 (3)  [1.1, 13] | 3.6 (2)  [0.62, 13] | 6.4 (3)  [1.7, 19] | 14.7 (24)  [9.8, 21] | 9.6 (32)  [6.7, 13] |
| *Very poor* |  | 2.0 (7)  [0.86, 4.2] |  | 0.0 (0)  [0.00, 6.6] | 0.0 (0)  [0.00, 8.0] | 0.0 (0)  [0.00, 9.4] | 4.3 (7)  [1.9, 9.0] | 2.1 (7)  [0.92, 4.4] |
| ***Autonomy regarding using the toilet*** | 353 |  | 332 |  |  |  |  |  |
| *Very good* |  | 33.1 (117)  [28, 38] |  | 44.9 (31)  [33, 57] | 45.5 (25)  [32, 59] | 31.9 (15)  [20, 47] | 24.8 (40)  [19, 32] | 33.4 (111)  [28, 39] |
| *Good* |  | 31.4 (111)  [27, 37] |  | 36.2 (25)  [25, 49] | 32.7 (18)  [21, 47] | 31.9 (15)  [20, 47] | 30.4 (49)  [24, 38] | 32.2 (107)  [27, 38] |
| *Fair* |  | 19.8 (70)  [16, 24] |  | 13.0 (9)  [6.5, 24] | 12.7 (7)  [5.7, 25] | 23.4 (11)  [13, 38] | 21.1 (34)  [15, 28] | 18.4 (61)  [14, 23] |
| *Poor* |  | 10.5 (37)  [7.6, 14] |  | 4.3 (3)  [1.1, 13] | 7.3 (4)  [2.4, 18] | 10.6 (5)  [4.0, 24] | 14.9 (24)  [10, 22] | 10.8 (36)  [7.8, 15] |
| *Very poor* |  | 5.1 (18)  [3.1, 8.1] |  | 1.4 (1)  [0.08, 8.9] | 1.8 (1)  [0.09, 11] | 2.1 (1)  [0.11, 13] | 8.7 (14)  [5.0, 14] | 5.1 (17)  [3.1, 8.2] |
| ***Autonomy regarding looking after own room*** | 352 |  | 331 |  |  |  |  |  |
| *Very good* |  | 36.9 (130)  [32, 42] |  | 50.7 (35)  [39, 63] | 50.0 (28)  [37, 63] | 29.8 (14)  [18, 45] | 30.2 (48)  [23, 38] | 37.8 (125)  [33, 43] |
| *Good* |  | 29.5 (104)  [25, 35] |  | 26.1 (18)  [17, 38] | 21.4 (12)  [12, 35] | 46.8 (22)  [32, 62] | 28.9 (46)  [22, 37] | 29.6 (98)  [25, 35] |
| *Fair* |  | 17.0 (60)  [13, 21] |  | 18.8 (13)  [11, 30] | 16.1 (9)  [8.1, 29] | 14.9 (7)  [6.7, 29] | 17.0 (27)  [12, 24] | 16.9 (56)  [13, 21] |
| *Poor* |  | 7.4 (26)  [5.0, 11] |  | 0.0 (0)  [0.00, 6.6] | 8.9 (5)  [3.3, 20] | 2.1 (1)  [0.11, 13] | 10.7 (17)  [6.5, 17] | 6.9 (23)  [4.6, 10] |
| *Very poor* |  | 9.1 (32)  [6.4, 13] |  | 4.3 (3)  [1.1, 13] | 3.6 (2)  [0.62, 13] | 6.4 (3)  [1.7, 19] | 13.2 (21)  [8.5, 20] | 8.8 (29)  [6.0, 12] |
| ***Autonomy regarding getting minor repairs done in own room*** | 353 |  | 332 |  |  |  |  |  |
| *Very good* |  | 18.7 (66)  [15, 23] |  | 29.0 (20)  [19, 41] | 17.9 (10)  [9.3, 31] | 21.3 (10)  [11, 36] | 14.4 (23)  [9.5, 21] | 19.0 (63)  [15, 24] |
| *Good* |  | 25.8 (91)  [21, 31] |  | 33.3 (23)  [23, 46] | 33.9 (19)  [22, 48] | 23.4 (11)  [13, 38] | 20.6 (33)  [15, 28] | 25.9 (86)  [21, 31] |
| *Fair* |  | 28.0 (99)  [23, 33] |  | 27.5 (19)  [18, 40] | 32.1 (18)  [21, 46] | 36.2 (17)  [23, 52] | 25.6 (41)  [19, 33] | 28.6 (95)  [24, 34] |
| *Poor* |  | 16.7 (59)  [13, 21] |  | 7.2 (5)  [2.7, 17] | 10.7 (6)  [4.4, 23] | 8.5 (4)  [2.8, 21] | 25.6 (41)  [19, 33] | 16.9 (56)  [13, 21] |
| *Very poor* |  | 10.8 (38)  [7.8, 15] |  | 2.9 (2)  [0.50, 11] | 5.4 (3)  [1.4, 16] | 10.6 (5)  [4.0, 24] | 13.8 (22)  [9.0, 20] | 9.6 (32)  [6.8, 13] |
| ***Autonomy regarding retreating to a private area*** | 351 |  | 331 |  |  |  |  |  |
| *Very good* |  | 19.7 (69)  [16, 24] |  | 29.4 (20)  [19, 42] | 23.2 (13)  [13, 37] | 21.3 (10)  [11, 36] | 14.4 (23)  [9.5, 21] | 19.9 (66)  [16, 25] |
| *Good* |  | 18.2 (64)  [14, 23] |  | 30.9 (21)  [21, 43] | 21.4 (12)  [12, 35] | 14.9 (7)  [6.7, 29] | 11.9 (19)  [7.5, 18] | 17.8 (59)  [14, 22] |
| *Fair* |  | 20.5 (72)  [16, 25] |  | 20.6 (14)  [12, 32] | 25.0 (14)  [15, 39] | 29.8 (14)  [18, 45] | 15.6 (25)  [11, 22] | 20.2 (67)  [16, 25] |
| *Poor* |  | 17.4 (61)  [14, 22] |  | 7.4 (5)  [2.7, 17] | 14.3 (8)  [6.8, 27] | 21.3 (10)  [11, 36] | 22.5 (36)  [16, 30] | 17.8 (59)  [14, 22] |
| *Very poor* |  | 24.2 (85)  [20, 29] |  | 11.8 (8)  [5.6, 22] | 16.1 (9)  [8.1, 29] | 12.8 (6)  [5.3, 26] | 35.6 (57)  [28, 44] | 24.2 (80)  [20, 29] |
| ***Autonomy regarding practicing personal traditions*** | 350 |  | 330 |  |  |  |  |  |
| *Very good* |  | 40.0 (140)  [35, 45] |  | 59.4 (41)  [47, 71] | 43.6 (24)  [31, 58] | 38.3 (18)  [25, 54] | 31.4 (50)  [24, 39] | 40.3 (133)  [35, 46] |
| *Good* |  | 29.4 (103)  [25, 35] |  | 23.2 (16)  [14, 35] | 29.1 (16)  [18, 43] | 42.6 (20)  [29, 58] | 28.3 (45)  [22, 36] | 29.4 (97)  [25, 35] |
| *Fair* |  | 18.6 (65)  [15, 23] |  | 11.6 (8)  [5.5, 22] | 20.0 (11)  [11, 33] | 10.6 (5)  [4.0, 24] | 22.0 (35)  [16, 29] | 17.9 (59)  [14, 23] |
| *Poor* |  | 6.0 (21)  [3.8, 9.2] |  | 2.9 (2)  [0.50, 11] | 5.5 (3)  [1.4, 16] | 2.1 (1)  [0.11, 13] | 8.8 (14)  [5.1, 15] | 6.1 (20)  [3.8, 9.4] |
| *Very poor* |  | 6.0 (21)  [3.8, 9.2] |  | 2.9 (2)  [0.50, 11] | 1.8 (1)  [0.09, 11] | 6.4 (3)  [1.7, 19] | 9.4 (15)  [5.6, 15] | 6.4 (21)  [4.1, 9.7] |
| ***Frequency of boredom*** | 351 |  | 331 |  |  |  |  |  |
| *Never* |  | 8.5 (30)  [5.9, 12] |  | 20.6 (14)  [12, 32] | 12.7 (7)  [5.7, 25] | 2.1 (1)  [0.11, 13] | 3.1 (5)  [1.1, 7.5] | 8.2 (27)  [5.5, 12] |
| *Rarely* |  | 10.5 (37)  [7.6, 14] |  | 22.1 (15)  [13, 34] | 18.2 (10)  [9.5, 31] | 8.5 (4)  [2.8, 21] | 3.1 (5)  [1.1, 7.5] | 10.3 (34)  [7.3, 14] |
| *Sometimes* |  | 23.6 (83)  [19, 29] |  | 30.9 (21)  [21, 43] | 30.9 (17)  [20, 45] | 25.5 (12)  [14, 41] | 16.8 (27)  [12, 24] | 23.3 (77)  [19, 28] |
| *Often* |  | 21.9 (77)  [18, 27] |  | 10.3 (7)  [4.6, 21] | 21.8 (12)  [12, 35] | 29.8 (14)  [18, 45] | 24.2 (39)  [18, 32] | 21.8 (72)  [18, 27] |
| *Always* |  | 35.3 (124)  [30, 41] |  | 16.2 (11)  [8.7, 28] | 16.4 (9)  [8.2, 29] | 34.0 (16)  [21, 49] | 52.8 (85)  [45, 61] | 36.6 (121)  [31, 42] |
| ***Frequency of feeling like being in prison*** | 348 |  | 328 |  |  |  |  |  |
| *Never* |  | 27.3 (95)  [23, 32] |  | 57.4 (39)  [45, 69] | 44.6 (25)  [32, 58] | 19.6 (9)  [9.9, 34] | 9.5 (15)  [5.6, 15] | 26.8 (88)  [22, 32] |
| *Rarely* |  | 6.9 (24)  [4.6, 10] |  | 8.8 (6)  [3.6, 19] | 12.5 (7)  [5.6, 25] | 0.0 (0)  [0.00, 9.6] | 5.1 (8)  [2.4, 10] | 6.4 (21)  [4.1, 9.8] |
| *Sometimes* |  | 20.7 (72)  [17, 25] |  | 22.1 (15)  [13, 34] | 23.2 (13)  [13, 37] | 26.1 (12)  [15, 41] | 17.1 (27)  [12, 24] | 20.4 (67)  [16, 25] |
| *Often* |  | 16.4 (57)  [13, 21] |  | 5.9 (4)  [1.9, 15] | 12.5 (7)  [5.6, 25] | 30.4 (14)  [18, 46] | 19.0 (30)  [13, 26] | 16.8 (55)  [13, 21] |
| *Always* |  | 28.7 (100)  [24, 34] |  | 5.9 (4)  [1.9, 15] | 7.1 (4)  [2.3, 18] | 23.9 (11)  [13, 39] | 49.4 (78)  [41, 57] | 29.6 (97)  [25, 35] |
| ***Room inspections*** | 376 |  | 331 |  |  |  |  |  |
| *No* |  | 30.6 (115)  [26, 36] |  | 24.3 (17)  [15, 36] | 35.8 (19)  [23, 50] | 46.8 (22)  [32, 62] | 28.0 (45)  [21, 36] | 31.1 (103)  [26, 36] |
| *Yes* |  | 69.4 (261)  [64, 74] |  | 75.7 (53)  [64, 85] | 64.2 (34)  [50, 77] | 53.2 (25)  [38, 68] | 72.0 (116)  [64, 79] | 68.9 (228)  [64, 74] |
| ***Living together with spouse in same accommodation*** | 41 |  | 41 |  |  |  |  |  |
| *Yes* |  | 92.7 (38)  [79, 98] |  | 100.0 (2)  [20, 100] | 66.7 (2)  [13, 98] | 100.0 (10)  [66, 100] | 92.3 (24)  [73, 99] | 92.7 (38)  [79, 98] |
| *No* |  | 7.3 (3)  [1.9, 21] |  | 0.0 (0)  [0.00, 80] | 33.3 (1)  [1.8, 87] | 0.0 (0)  [0.00, 34] | 7.7 (2)  [1.3, 27] | 7.3 (3)  [1.9, 21] |
| ***Living together with children in same accommodation*** | 73 |  | 73 |  |  |  |  |  |
| *Living with all children in the same accommodation* |  | 75.3 (55)  [64, 84] |  | 66.7 (6)  [31, 91] | 66.7 (4)  [24, 94] | 86.7 (13)  [58, 98] | 74.4 (32)  [59, 86] | 75.3 (55)  [64, 84] |
| *Living with some but not all children in the same accommodation* |  | 8.2 (6)  [3.4, 18] |  | 11.1 (1)  [0.58, 49] | 0.0 (0)  [0.00, 48] | 0.0 (0)  [0.00, 25] | 11.6 (5)  [4.4, 26] | 8.2 (6)  [3.4, 18] |
| *Living with none child in the same accommodation* |  | 16.4 (12)  [9.1, 27] |  | 22.2 (2)  [3.9, 60] | 33.3 (2)  [6.0, 76] | 13.3 (2)  [2.3, 42] | 14.0 (6)  [5.8, 29] | 16.4 (12)  [9.1, 27] |
| ***Central food provision (e.g. catering)*** | 410 |  | 343 |  |  |  |  |  |
| *Yes* |  | 8.0 (33)  [5.7, 11] |  | 8.3 (6)  [3.4, 18] | 7.1 (4)  [2.3, 18] | 3.9 (2)  [0.68, 15] | 11.6 (19)  [7.3, 18] | 9.0 (31)  [6.3, 13] |
| *No* |  | 92.0 (377)  [89, 94] |  | 91.7 (66)  [82, 97] | 92.9 (52)  [82, 98] | 96.1 (49)  [85, 99] | 88.4 (145)  [82, 93] | 91.0 (312)  [87, 94] |
| ***Food quality*** | 31 |  | 30 |  |  |  |  |  |
| *Very good* |  | 12.9 (4)  [4.2, 31] |  | 16.7 (1)  [0.88, 64] | 50.0 (2)  [15, 85] | 0.0 (0)  [0.00, 80] | 5.6 (1)  [0.29, 29] | 13.3 (4)  [4.4, 32] |
| *Fairly good* |  | 19.4 (6)  [8.1, 38] |  | 66.7 (4)  [24, 94] | 0.0 (0)  [0.00, 60] | 50.0 (1)  [9.5, 91] | 5.6 (1)  [0.29, 29] | 20.0 (6)  [8.4, 39] |
| *Rather bad* |  | 32.3 (10)  [17, 51] |  | 0.0 (0)  [0.00, 48] | 25.0 (1)  [1.3, 78] | 50.0 (1)  [9.5, 91] | 44.4 (8)  [22, 69] | 33.3 (10)  [18, 53] |
| *Very bad* |  | 35.5 (11)  [20, 55] |  | 16.7 (1)  [0.88, 64] | 25.0 (1)  [1.3, 78] | 0.0 (0)  [0.00, 80] | 44.4 (8)  [22, 69] | 33.3 (10)  [18, 53] |
| ***Food quantity*** | 30 |  | 29 |  |  |  |  |  |
| *Very good* |  | 16.7 (5)  [6.3, 35] |  | 0.0 (0)  [0.00, 54] | 50.0 (2)  [15, 85] | 50.0 (1)  [9.5, 91] | 11.1 (2)  [1.9, 36] | 17.2 (5)  [6.5, 36] |
| *Fairly good* |  | 43.3 (13)  [26, 62] |  | 100.0 (5)  [46, 100] | 0.0 (0)  [0.00, 60] | 50.0 (1)  [9.5, 91] | 38.9 (7)  [18, 64] | 44.8 (13)  [27, 64] |
| *Rather bad* |  | 23.3 (7)  [11, 43] |  | 0.0 (0)  [0.00, 54] | 25.0 (1)  [1.3, 78] | 0.0 (0)  [0.00, 80] | 27.8 (5)  [11, 54] | 20.7 (6)  [8.7, 40] |
| *Very bad* |  | 16.7 (5)  [6.3, 35] |  | 0.0 (0)  [0.00, 54] | 25.0 (1)  [1.3, 78] | 0.0 (0)  [0.00, 80] | 22.2 (4)  [7.4, 48] | 17.2 (5)  [6.5, 36] |
