# Supplementary material for: Perceived housing quality and emotional distress among refugees living in camps: a cross-sectional study in Eastern Germany
Source: BMC Public Health. 2026 Nov 14;26:3381. doi: 10.1186/s12889-026-30396-8 (PMC13770332; doi:10.1186/s12889-026-30396-8)
Supplement: Supplementary file 3 [file 12889_2026_30396_MOESM3_ESM.docx]

## Table 1: Sensitivity analyses

|  | **S1: Main model with non-imputed data** | | **S2: Main model without subjective health status and interpreters’ dependency** | |
| --- | --- | --- | --- | --- |
| **Variable** | **β** | **95% CI** | **β** | **95% CI** |
| **Crowding (camp)** (ref. *Adequate*) |  |  |  |  |
| *Tolerable* | -0.66 | [-3.5, 2.2] | 0.31 | [-2.58, 3.19] |
| *(Extremely) Intolerable* | -0.28 | [-3.4, 2.8] | 1.25 | [-1.90, 4.39] |
| **Room size** *Poor* (ref. *Good*) | 2.1 | [-0.44, 4.6] | 3.00 | [0.46, 5.54] |
| **Frequent transfers** *Yes* (ref. *No*) | 1.1 | [-1.3, 3.6] | 0.83 | [-1.78, 3.43] |
| **Safety (room)** *Unsafe* (ref. *Safe*) | 3.7 | [0.65, 6.7] | 3.66 | [0.56, 6.76] |
| **Lighting conditions (room)** *Poor* (ref. *Good*) | 5.3 | [2.1, 8.4] | 5.25 | [1.87, 8.63] |
| **Region** *Rural* (ref. *urban)* | 1.2 | [-1.0, 3.4] | 1.43 | [-0.94, 3.80] |
| **Autonomy** (mean score) | 3.0 | [1.6, 4.4] | 3.37 | [1.90, 4.84] |
| **Frequency of feeling bored** *High* (ref. *Low*) | 3.3 | [0.55, 6.0] | 5.33 | [2.61, 8.05] |
| **Frequency of feeling imprisoned** *High* (ref. *Low*) | 4.9 | [1.9, 7.9] | 4.75 | [1.72, 7.78] |

*Notes.*

S1 was adjusted for age (years), gender (ref. *male*), country of origin (ref. *Syria*), marital status (ref. *single*), length of stay in Germany (months), dependency on interpreters (ref. *no*), and subjective general health status (ref. *excellent/good/very good*).

S2 excluded dependency on interpreters and subjective general health status. Estimates for S2 are pooled across 10 multiple imputed datasets using Rubin’s rule.
