# Supplementary material for: Perceived housing quality and emotional distress among refugees living in camps: a cross-sectional study in Eastern Germany
Source: BMC Public Health. 2026 Nov 14;26:3381. doi: 10.1186/s12889-026-30396-8 (PMC13770332; doi:10.1186/s12889-026-30396-8)
Supplement: Supplementary file 4 [file 12889_2026_30396_MOESM4_ESM.docx]

Main model with imputation set 1

Main model with imputation set 5

Main model with imputation set 10

RHS-13 sum score Histogram

| Imputation | Breusch.Pagan.p | Breusch-Pagan p |
| --- | --- | --- |
| 1 | 0.011523722 | 0.012 |
| 2 | 0.010897758 | 0.011 |
| 3 | 0.042732575 | 0.043 |
| 4 | 0.041706495 | 0.042 |
| 5 | 0.003117949 | 0.003 |
| 6 | 0.045947398 | 0.046 |
| 7 | 0.033216835 | 0.033 |
| 8 | 0.026187548 | 0.026 |
| 9 | 0.062019163 | 0.062 |
| 10 | 0.018539310 | 0.019 |

Main model with robust SE

| Predictor | Estimate | Robust SE (HC3) | 95% CI | p |
| --- | --- | --- | --- | --- |
| (Intercept) | -5.07 | 2.74 | [-10.46; 0.33] | 0.065 |
| accommodationCrowding_combinedTolerable | -0.84 | 1.49 | [-3.77; 2.09] | 0.573 |
| accommodationCrowding_combined(Extremely)Untolerable | 0.05 | 1.61 | [-3.12; 3.22] | 0.975 |
| roomSize_combinedPoor | 2.51 | 1.33 | [-0.12; 5.13] | 0.061 |
| frequentTransfersYes | 0.96 | 1.21 | [-1.42; 3.34] | 0.428 |
| roomSafety_combinedUnsafe | 3.02 | 1.49 | [0.09; 5.95] | 0.043 |
| roomLight_combinedPoor | 5.01 | 1.68 | [1.71; 8.32] | 0.003 |
| regionRural | 1.43 | 1.13 | [-0.80; 3.66] | 0.209 |
| autonomyIndex | 2.90 | 0.71 | [1.50; 4.31] | <0.001 |
| feelingBored_combinedHigh | 3.22 | 1.41 | [0.44; 5.99] | 0.023 |
| feelingPrison_combinedHigh | 4.69 | 1.49 | [1.76; 7.62] | 0.002 |
| age | 0.08 | 0.06 | [-0.05; 0.20] | 0.235 |
| genderFemale | 3.36 | 1.68 | [0.05; 6.67] | 0.047 |
| origin_combinedAfghanistan | 5.02 | 1.56 | [1.95; 8.09] | 0.001 |
| origin_combinedOther African countries | 3.48 | 2.10 | [-0.65; 7.60] | 0.098 |
| origin_combinedOther | 5.05 | 1.32 | [2.45; 7.66] | <0.001 |
| familyStatus_combinedMarried | 2.74 | 1.32 | [0.15; 5.33] | 0.038 |
| familyStatus_combinedDivorced/Widowed | 1.37 | 2.69 | [-3.93; 6.66] | 0.611 |
| residenceDuration | 0.02 | 0.02 | [-0.03; 0.07] | 0.518 |
| interpretersDependencyYes | 0.96 | 1.35 | [-1.69; 3.61] | 0.476 |
| subjectiveHealth_combinedPoor | 8.55 | 1.32 | [5.96; 11.14] | <0.001 |

Single indicator models with robust SE

| **Characteristic** | **Beta** | **95% CI** | **p-value** |
| --- | --- | --- | --- |
| Crowding (room) |  |  |  |
| Adequate | — | — |  |
| Tolerable | 2.5 | -1.4, 6.3 | 0.208 |
| (Extremely)Untolerable | 5.1 | 1.6, 8.6 | 0.004 |
| Crowding (accommodation) |  |  |  |
| Adequate | — | — |  |
| Tolerable | 0.23 | -3.2, 3.7 | 0.895 |
| (Extremely)Untolerable | 6.1 | 2.6, 9.5 | <0.001 |
| Crowding (kitchen) |  |  |  |
| Adequate | — | — |  |
| Tolerable | 6.7 | 3.2, 10 | <0.001 |
| (Extremely)Untolerable | 10 | 7.0, 13 | <0.001 |
| Crowding (shower) |  |  |  |
| Adequate | — | — |  |
| Tolerable | 3.5 | -1.2, 8.3 | 0.142 |
| (Extremely)Untolerable | 7.5 | 2.9, 12 | 0.001 |
| Crowding (toilet) |  |  |  |
| Adequate | — | — |  |
| Tolerable | 3.0 | -1.4, 7.4 | 0.185 |
| (Extremely)Untolerable | 6.9 | 2.7, 11 | 0.001 |
| Room size |  |  |  |
| Good | — | — |  |
| Poor | 7.0 | 4.4, 9.6 | <0.001 |
| Possibility to store personal items |  |  |  |
| Yes | — | — |  |
| No | 0.37 | -2.4, 3.1 | 0.794 |
| Possibility to lock own room |  |  |  |
| Yes | — | — |  |
| No | 0.45 | -4.9, 5.8 | 0.870 |
| Frequent transfers |  |  |  |
| No | — | — |  |
| Yes | 3.9 | 1.3, 6.5 | 0.004 |
| Safety (room) |  |  |  |
| Safe | — | — |  |
| Unsafe | 8.7 | 5.7, 12 | <0.001 |
| Safety (accommodation) |  |  |  |
| Safe | — | — |  |
| Unsafe | 7.5 | 4.5, 11 | <0.001 |
| Safety (shower) |  |  |  |
| Safe | — | — |  |
| Unsafe | 6.9 | 4.0, 9.8 | <0.001 |
| Safety (toilet) |  |  |  |
| Safe | — | — |  |
| Unsafe | 7.0 | 3.9, 10 | <0.001 |
| Lighting conditions (room) |  |  |  |
| Good | — | — |  |
| Poor | 8.9 | 5.6, 12 | <0.001 |
| Hot water supply (accommodation) |  |  |  |
| Good | — | — |  |
| Poor | 3.4 | -1.3, 8.2 | 0.155 |
| Hygiene (kitchen) |  |  |  |
| Good | — | — |  |
| Poor | 6.3 | 3.6, 9.0 | <0.001 |
| Hygiene (shower) |  |  |  |
| Good | — | — |  |
| Poor | 5.2 | 2.4, 7.9 | <0.001 |
| Hygiene (toilet) |  |  |  |
| Good | — | — |  |
| Poor | 5.9 | 3.4, 8.4 | <0.001 |
| Region |  |  |  |
| Urban | — | — |  |
| Rural | 2.7 | 0.15, 5.2 | 0.038 |
| Indoor autonomy (mean score) | 5.5 | 4.2, 6.9 | <0.001 |
| Frequency of feeling like being in prison |  |  |  |
| Low | — | — |  |
| High | 11 | 8.1, 13 | <0.001 |
| Frequency of boredom |  |  |  |
| Low | — | — |  |
| High | 8.1 | 5.5, 11 | <0.001 |
| Room inspections |  |  |  |
| No | — | — |  |
| Yes | -0.28 | -3.0, 2.5 | 0.843 |
| Abbreviation: CI = Confidence Interval | | | |

Sensitivity model 1 with robust SE

| **Predictor** | **Estimate** | **SE** | **95% CI** | **p** |
| --- | --- | --- | --- | --- |
| (Intercept) | -4.93 | 2.96 | [-10.76; 0.89] | 0.097 |
| accommodationCrowding_combinedTolerable | -0.66 | 1.58 | [-3.78; 2.46] | 0.677 |
| accommodationCrowding_combined(Extremely)Untolerable | -0.28 | 1.75 | [-3.73; 3.17] | 0.873 |
| roomSize_combinedPoor | 2.08 | 1.43 | [-0.73; 4.89] | 0.145 |
| frequentTransfersYes | 1.13 | 1.25 | [-1.33; 3.59] | 0.368 |
| roomSafety_combinedUnsafe | 3.67 | 1.57 | [0.57; 6.76] | 0.020 |
| roomLight_combinedPoor | 5.26 | 1.73 | [1.85; 8.66] | 0.003 |
| regionRural | 1.20 | 1.17 | [-1.11; 3.50] | 0.308 |
| autonomyIndex | 2.99 | 0.75 | [1.52; 4.45] | <0.001 |
| feelingBored_combinedHigh | 3.25 | 1.49 | [0.32; 6.18] | 0.030 |
| feelingPrison_combinedHigh | 4.94 | 1.57 | [1.85; 8.02] | 0.002 |
| age | 0.07 | 0.07 | [-0.07; 0.21] | 0.307 |
| genderFemale | 4.38 | 1.79 | [0.85; 7.91] | 0.015 |
| origin_combinedAfghanistan | 5.31 | 1.69 | [1.98; 8.65] | 0.002 |
| origin_combinedOther African countries | 3.26 | 2.19 | [-1.05; 7.56] | 0.138 |
| origin_combinedOther | 5.41 | 1.38 | [2.70; 8.12] | <0.001 |
| familyStatus_combinedMarried | 2.57 | 1.37 | [-0.12; 5.26] | 0.061 |
| familyStatus_combinedDivorced/Widowed | 1.53 | 2.90 | [-4.19; 7.25] | 0.598 |
| residenceDuration | 0.02 | 0.03 | [-0.04; 0.07] | 0.519 |
| interpretersDependencyYes | 0.37 | 1.41 | [-2.41; 3.15] | 0.793 |
| subjectiveHealth_combinedPoor | 8.36 | 1.39 | [5.62; 11.11] | <0.001 |

| Sensitivity model 2 with robust SE |  |  |  |  |
| --- | --- | --- | --- | --- |
| **Predictor** | **Estimate** | **SE** | **95% CI** | **p** |
| (Intercept) | -6.48 | 3.06 | [-12.54; -0.41] | 0.037 |
| accommodationCrowding_combinedTolerable | 0.31 | 1.55 | [-2.75; 3.37] | 0.844 |
| accommodationCrowding_combined(Extremely)Untolerable | 1.25 | 1.71 | [-2.11; 4.60] | 0.465 |
| roomSize_combinedPoor | 3.00 | 1.39 | [0.27; 5.74] | 0.032 |
| frequentTransfersYes | 0.83 | 1.39 | [-1.91; 3.56] | 0.552 |
| roomSafety_combinedUnsafe | 3.66 | 1.61 | [0.49; 6.83] | 0.024 |
| roomLight_combinedPoor | 5.25 | 1.77 | [1.76; 8.74] | 0.003 |
| regionRural | 1.43 | 1.26 | [-1.06; 3.92] | 0.259 |
| autonomyIndex | 3.37 | 0.79 | [1.82; 4.92] | <0.001 |
| feelingBored_combinedHigh | 5.33 | 1.44 | [2.50; 8.16] | <0.001 |
| feelingPrison_combinedHigh | 4.75 | 1.56 | [1.68; 7.82] | 0.003 |
| age | 0.16 | 0.08 | [0.00; 0.32] | 0.049 |
| genderFemale | 4.13 | 1.72 | [0.75; 7.51] | 0.017 |
| origin_combinedAfghanistan | 6.07 | 1.77 | [2.58; 9.55] | <0.001 |
| origin_combinedOther African countries | 5.27 | 2.09 | [1.15; 9.39] | 0.012 |
| origin_combinedOther | 5.84 | 1.44 | [3.01; 8.67] | <0.001 |
| familyStatus_combinedMarried | 2.71 | 1.49 | [-0.22; 5.64] | 0.070 |
| familyStatus_combinedDivorced/Widowed | -0.14 | 2.93 | [-5.91; 5.64] | 0.963 |
| residenceDuration | 0.01 | 0.03 | [-0.05; 0.07] | 0.747 |
